# Supplementary figures and images for: Synergistic stabilization of microtubules by BUB-1, HCP-1, and CLS-2 controls microtubule pausing and meiotic spindle assembly
Source: eLife. 2023 Feb 17;12:e82579. doi: 10.7554/eLife.82579 (PMC10005782; doi:10.7554/eLife.82579)

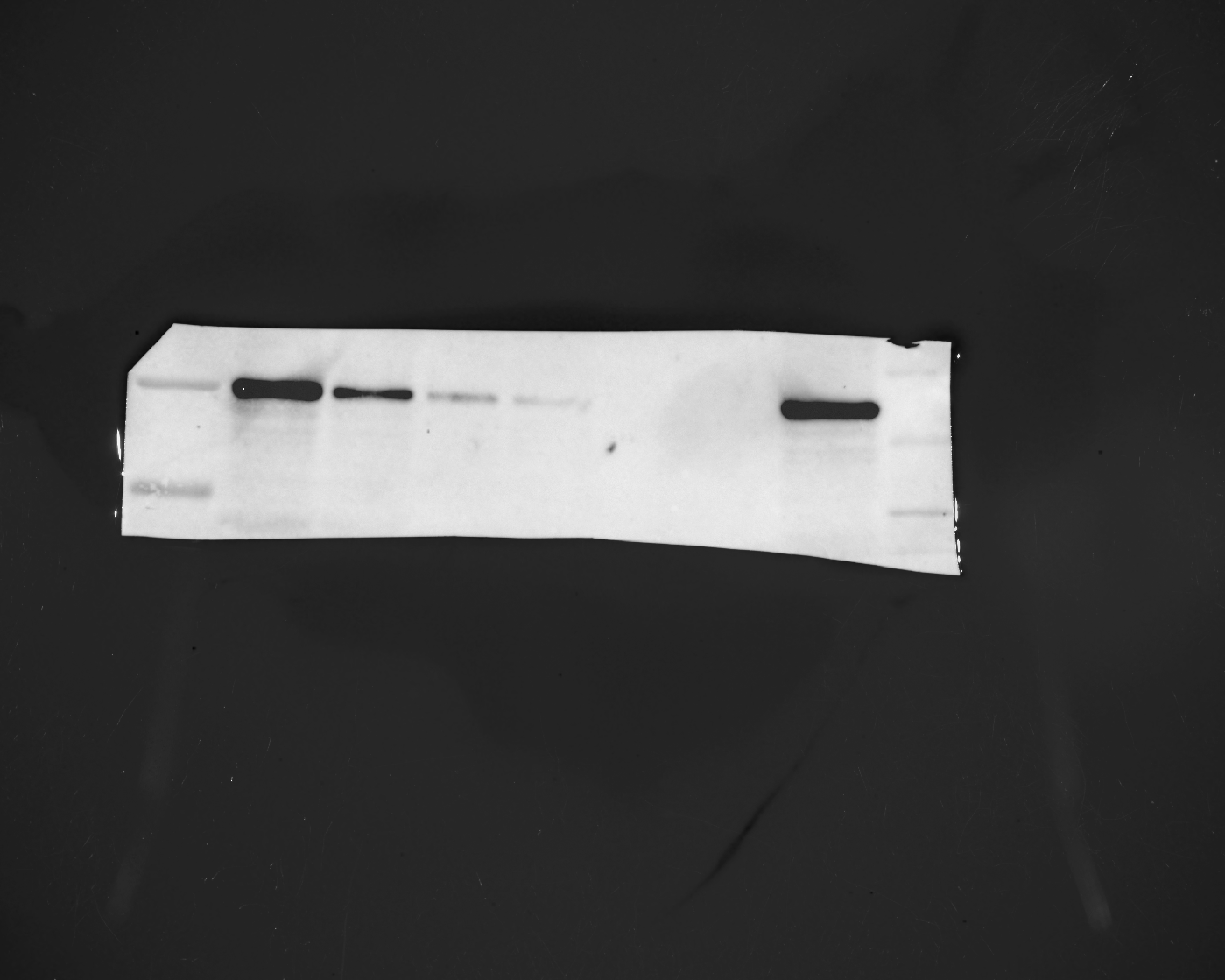

Supplement: Figure 1—figure supplement 1—source data 1. — Folder containing raw images and uncropped annotated images of Western blot of BUB-1 and tubulin in full-protein extracts of wild type and bub-3(ok3437) (bub-3∆) mutant worms. [file elife-82579-fig1-figsupp1-data1.zip › WB_anti-TUBa_CompositeSUM_8bit.tif]

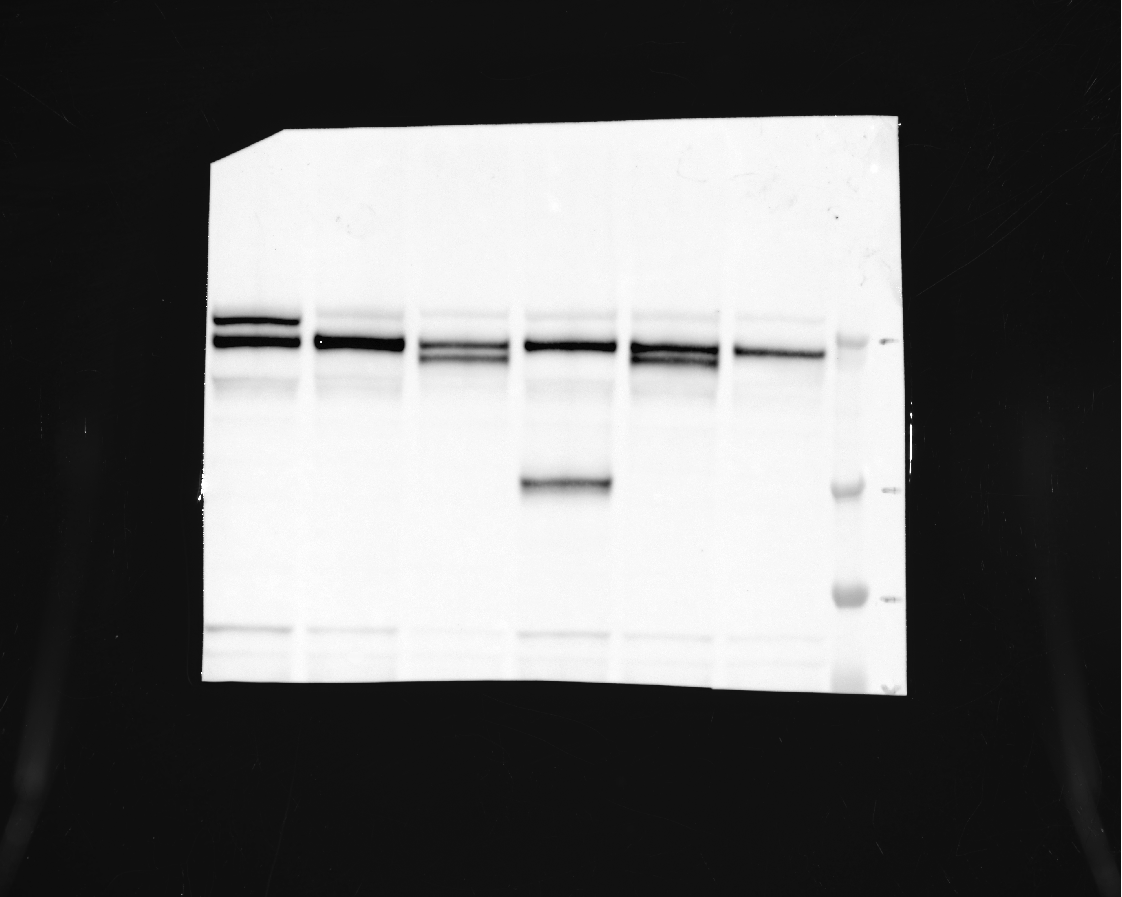

Supplement: Figure 2—figure supplement 1—source data 2. — Raw images and uncropped annotated images of western blots of GFP::HCP-1 fusion protein variants in full-protein worm extracts. [file elife-82579-fig2-figsupp1-data2.zip › Figure 2—figure supplement 1—source data 2/WB1_antiHCP1_Composite.tif]

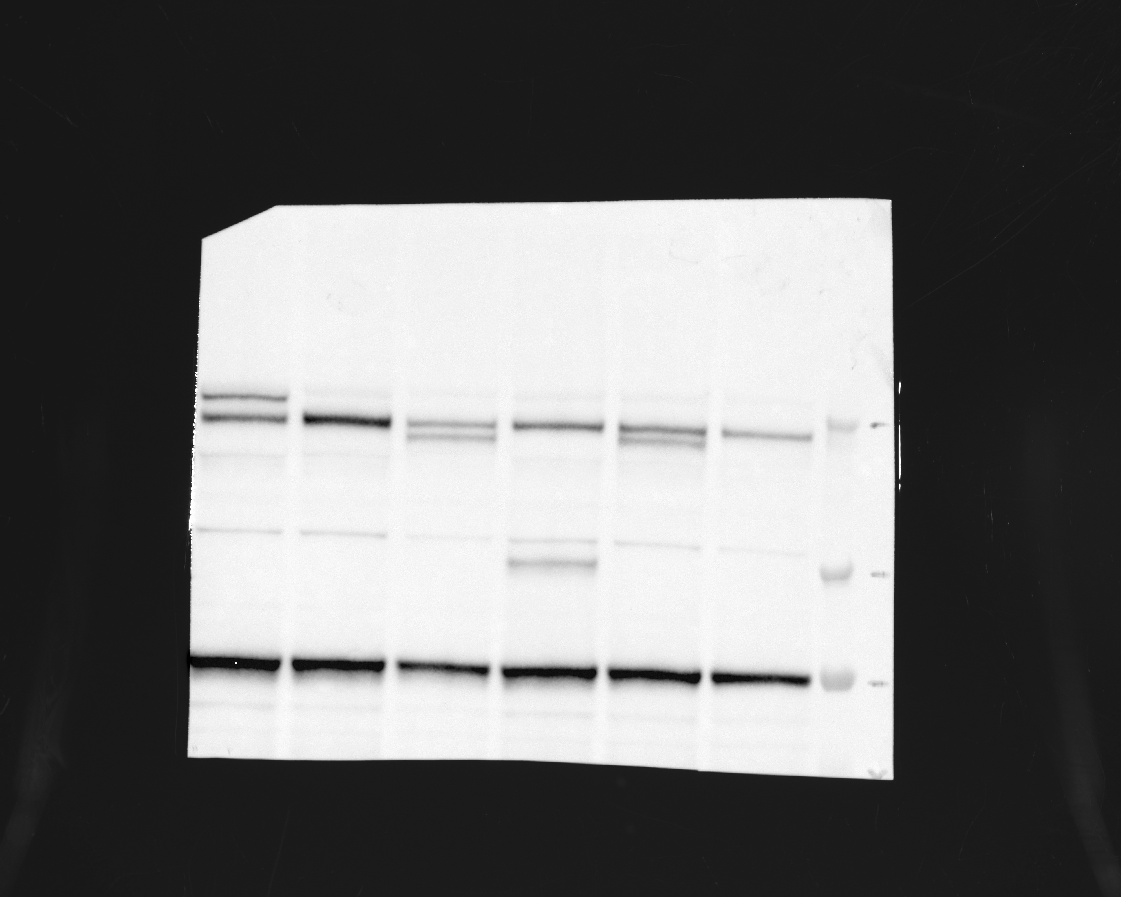

Supplement: Figure 2—figure supplement 1—source data 2. — Raw images and uncropped annotated images of western blots of GFP::HCP-1 fusion protein variants in full-protein worm extracts. [file elife-82579-fig2-figsupp1-data2.zip › Figure 2—figure supplement 1—source data 2/WB1_antiKLP7_Composite.tif]

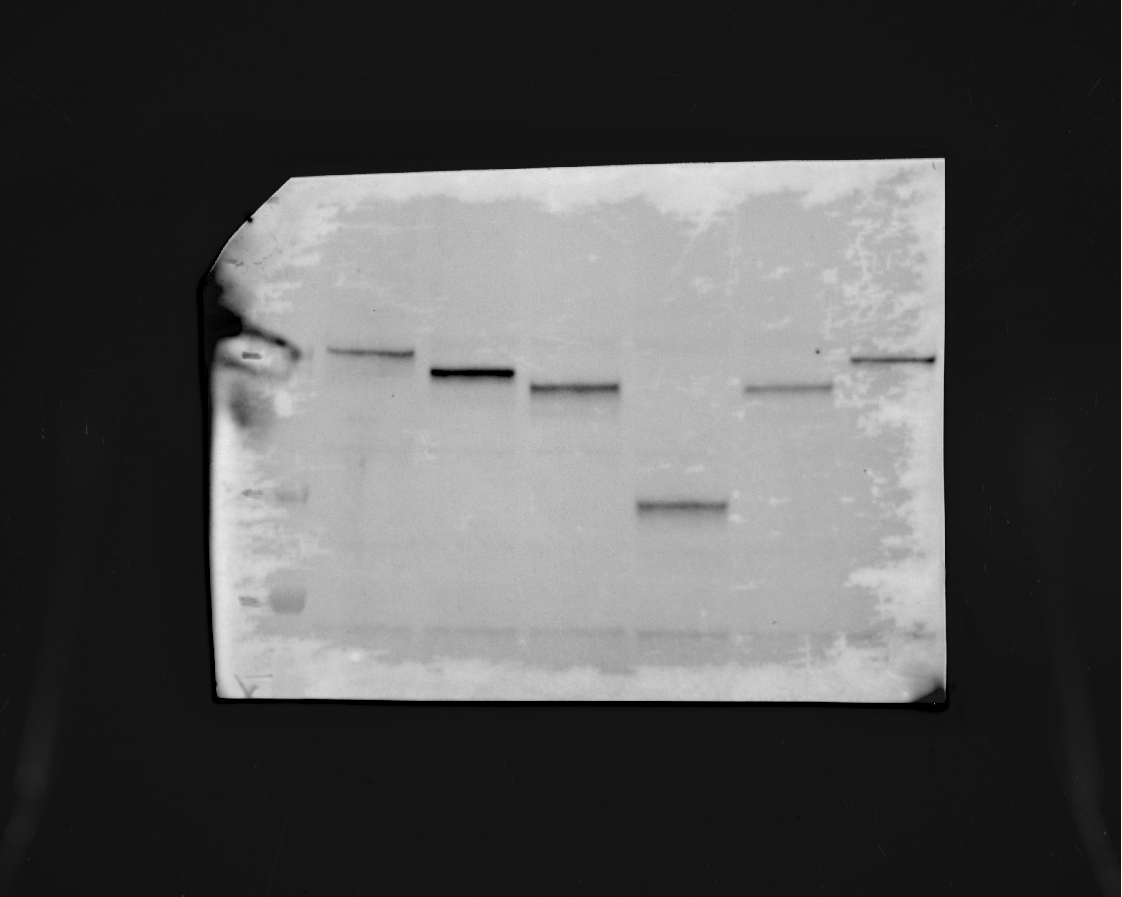

Supplement: Figure 2—figure supplement 1—source data 2. — Raw images and uncropped annotated images of western blots of GFP::HCP-1 fusion protein variants in full-protein worm extracts. [file elife-82579-fig2-figsupp1-data2.zip › Figure 2—figure supplement 1—source data 2/WB2_antiGFP_Composite.tif]

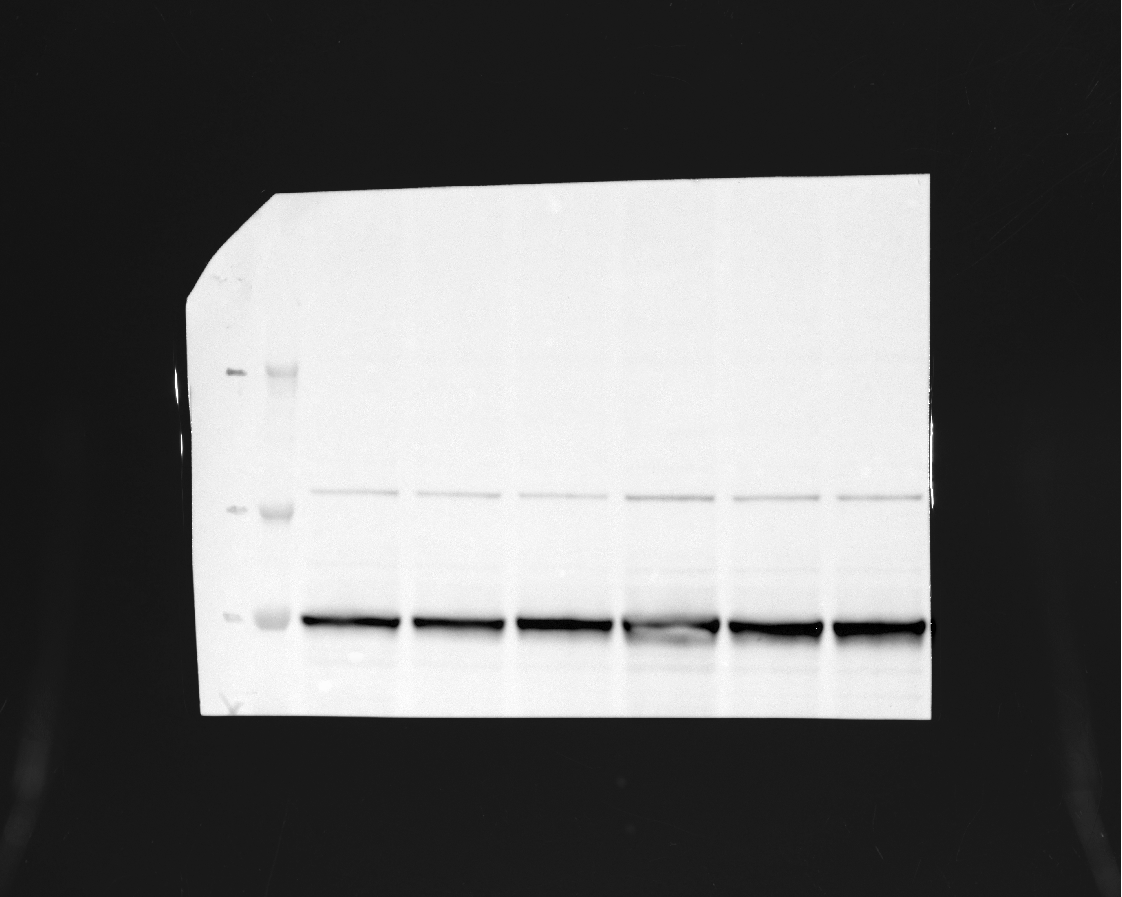

Supplement: Figure 2—figure supplement 1—source data 2. — Raw images and uncropped annotated images of western blots of GFP::HCP-1 fusion protein variants in full-protein worm extracts. [file elife-82579-fig2-figsupp1-data2.zip › Figure 2—figure supplement 1—source data 2/WB2_antiKLP7_Composite.tif]

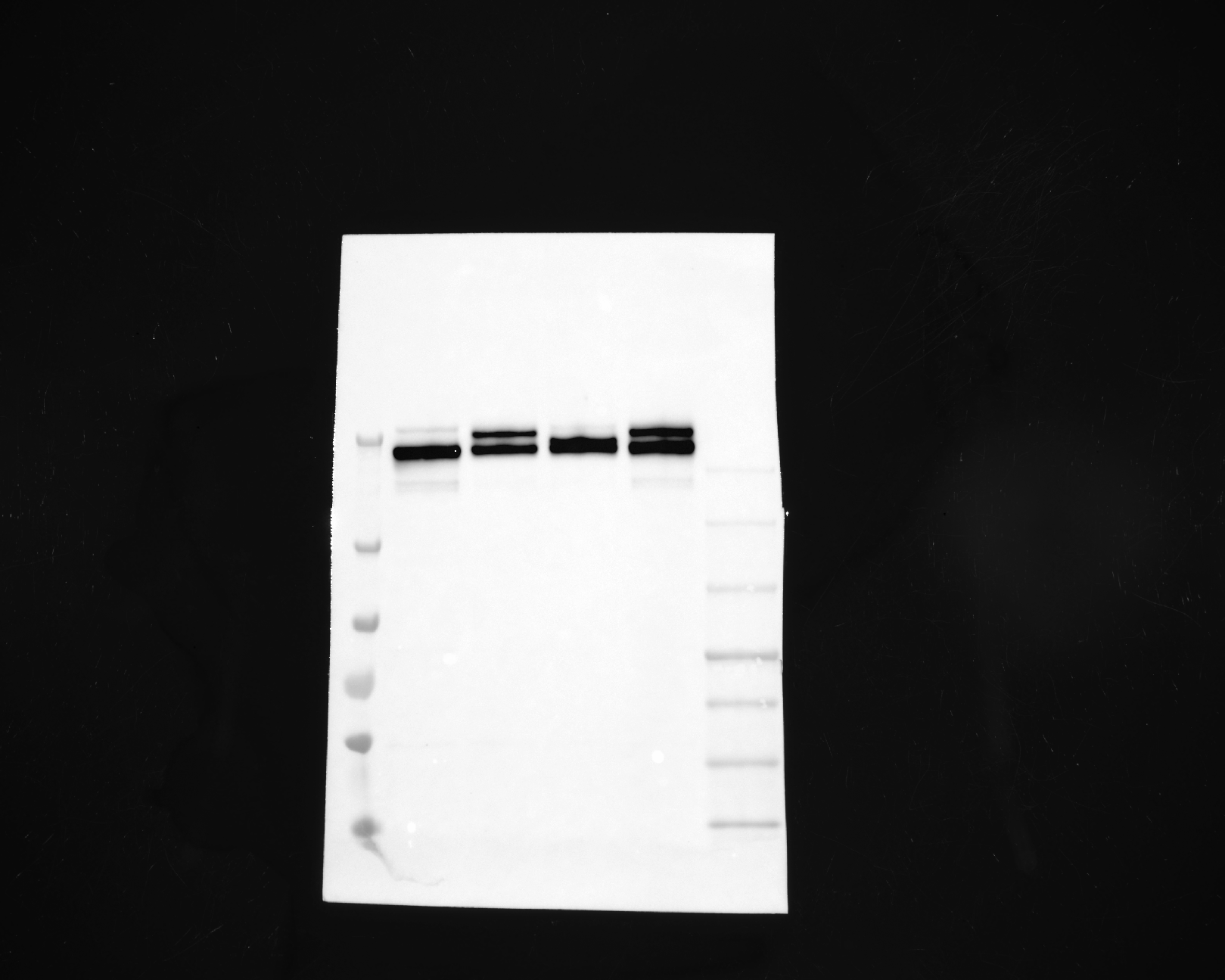

Supplement: Figure 2—figure supplement 1—source data 2. — Raw images and uncropped annotated images of western blots of GFP::HCP-1 fusion protein variants in full-protein worm extracts. [file elife-82579-fig2-figsupp1-data2.zip › Figure 2—figure supplement 1—source data 2/WB3_antiHCP1_SUM_Composite_8bit.tif]

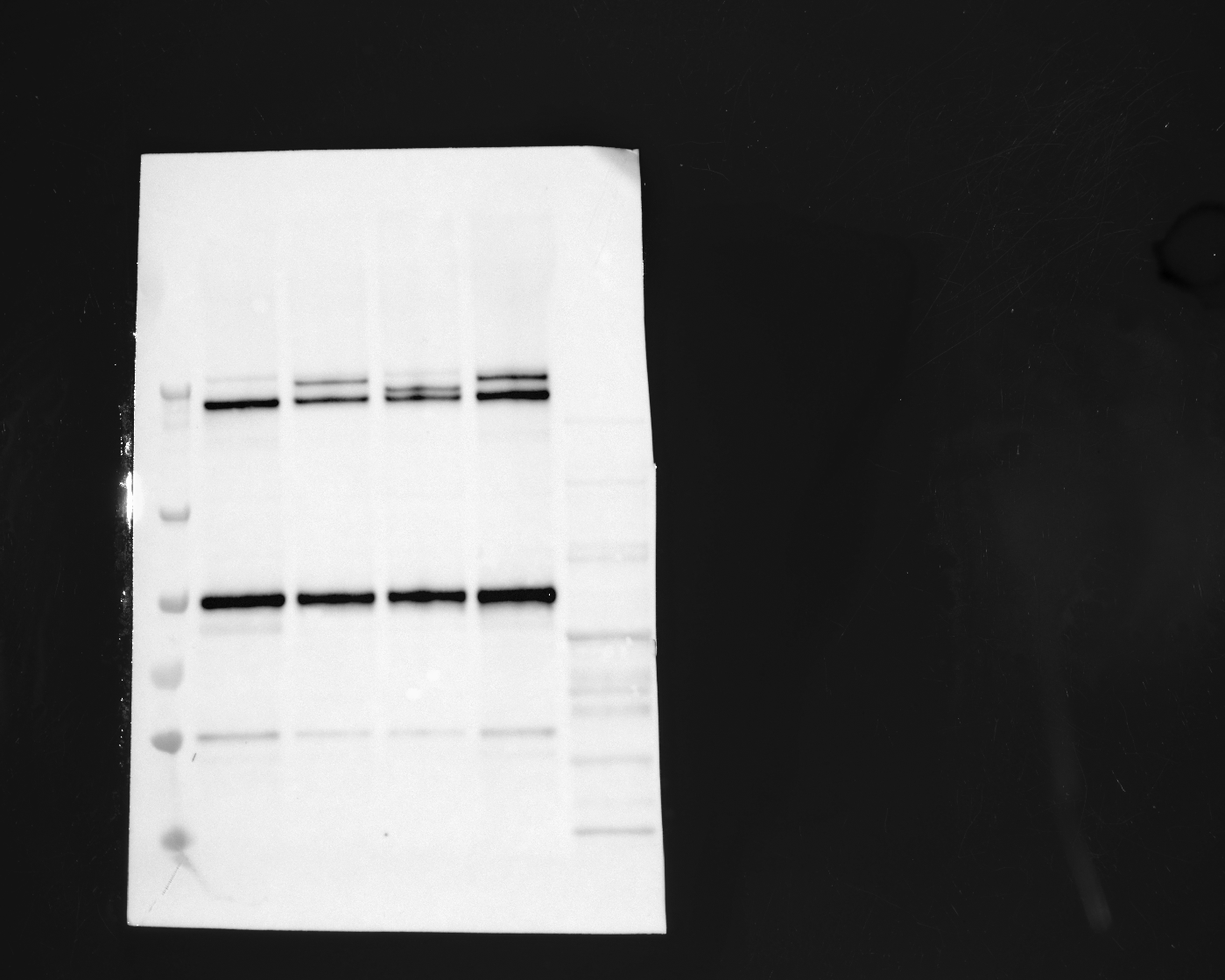

Supplement: Figure 2—figure supplement 1—source data 2. — Raw images and uncropped annotated images of western blots of GFP::HCP-1 fusion protein variants in full-protein worm extracts. [file elife-82579-fig2-figsupp1-data2.zip › Figure 2—figure supplement 1—source data 2/WB3_antiKLP7_Composite_8bits.tif]

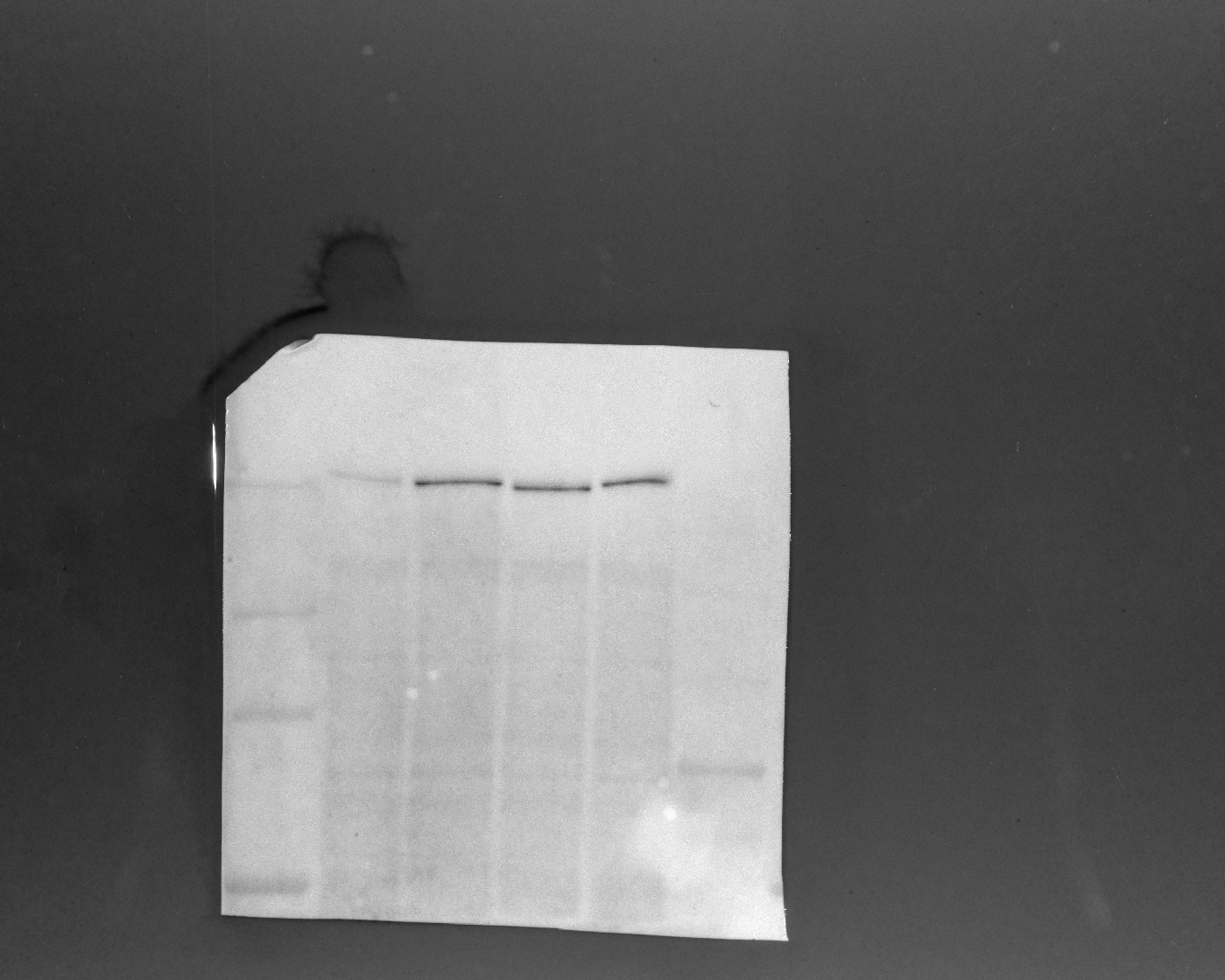

Supplement: Figure 2—figure supplement 1—source data 2. — Raw images and uncropped annotated images of western blots of GFP::HCP-1 fusion protein variants in full-protein worm extracts. [file elife-82579-fig2-figsupp1-data2.zip › Figure 2—figure supplement 1—source data 2/WB4_anti-HCP1_SUM_Composite_8bits.tif]

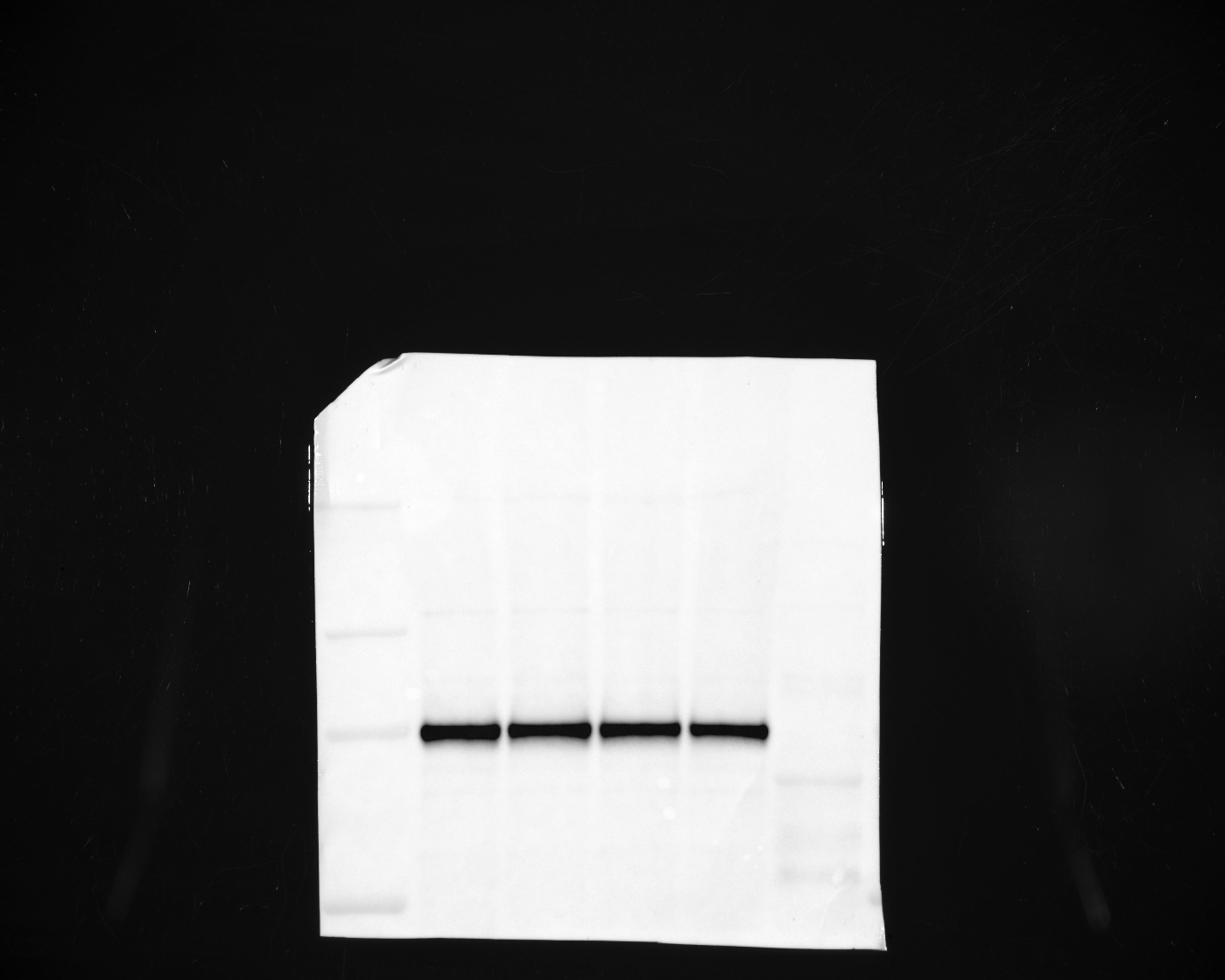

Supplement: Figure 2—figure supplement 1—source data 2. — Raw images and uncropped annotated images of western blots of GFP::HCP-1 fusion protein variants in full-protein worm extracts. [file elife-82579-fig2-figsupp1-data2.zip › Figure 2—figure supplement 1—source data 2/WB4_anti-KLP7_SUM_Composite-1_8bits.tif]

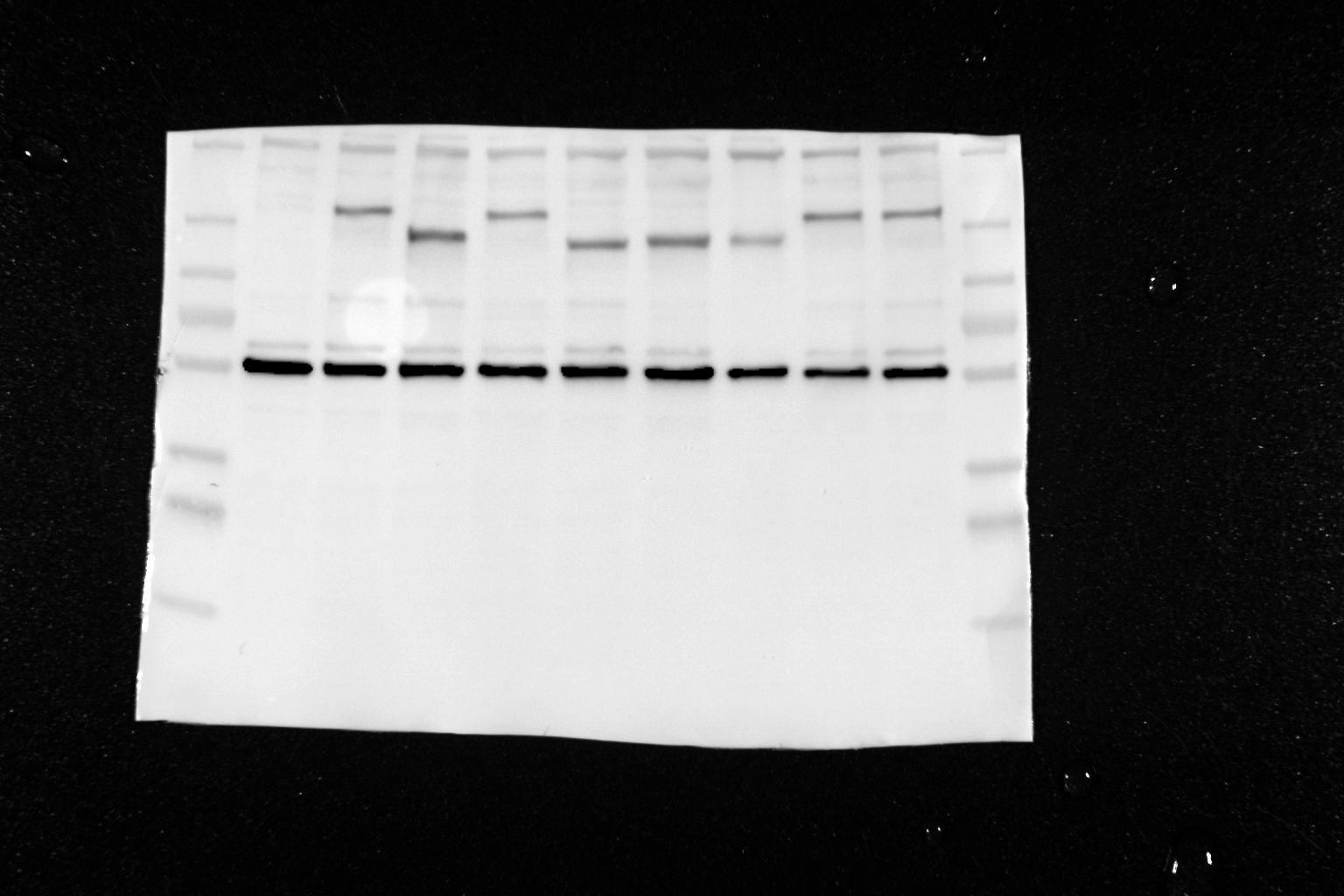

Supplement: Figure 5—figure supplement 1—source data 1. — Raw images and uncropped annotated image of western blots of CLS-2::GFP fusion protein variants in full-protein worm extracts. [file elife-82579-fig5-figsupp1-data1.zip › Figure 5—figure supplement 1—source data 1/WB1_antiGFP_antiTub_composite.tif]

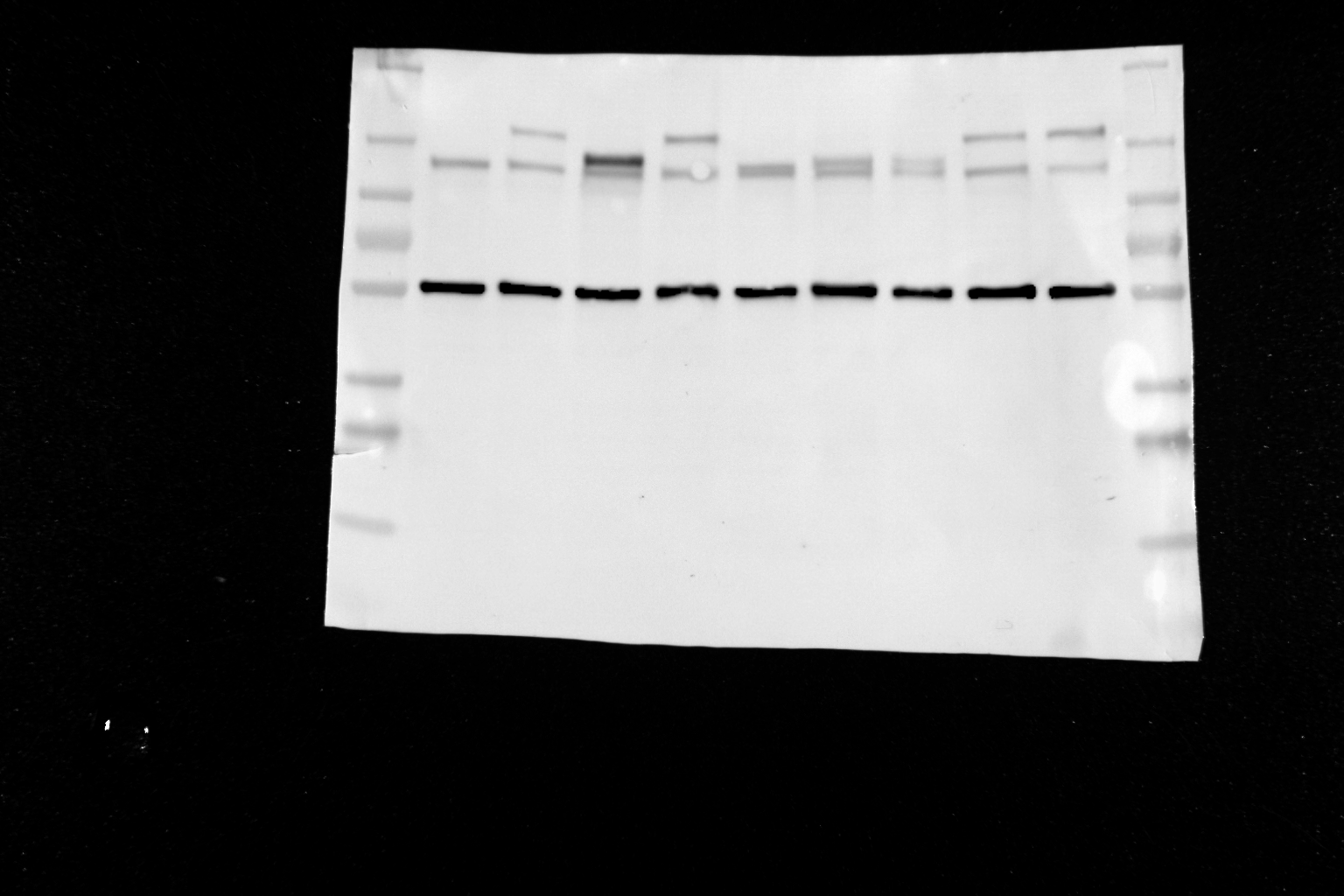

Supplement: Figure 5—figure supplement 1—source data 1. — Raw images and uncropped annotated image of western blots of CLS-2::GFP fusion protein variants in full-protein worm extracts. [file elife-82579-fig5-figsupp1-data1.zip › Figure 5—figure supplement 1—source data 1/WB2_antiCLS-2_antiTub_composite.tif]

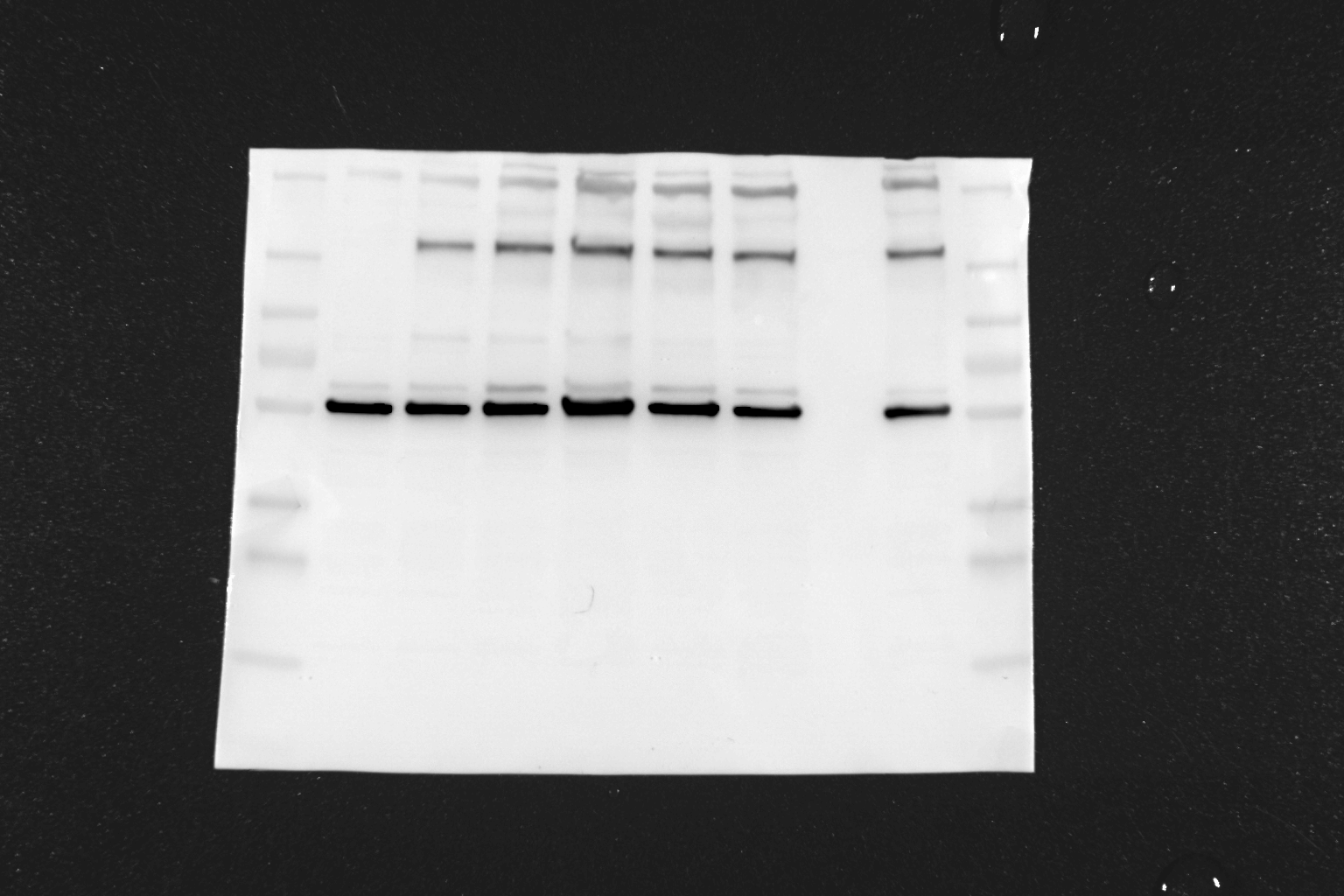

Supplement: Figure 5—figure supplement 1—source data 1. — Raw images and uncropped annotated image of western blots of CLS-2::GFP fusion protein variants in full-protein worm extracts. [file elife-82579-fig5-figsupp1-data1.zip › Figure 5—figure supplement 1—source data 1/WB3_antiGFP_antiTub_Composite.tif]

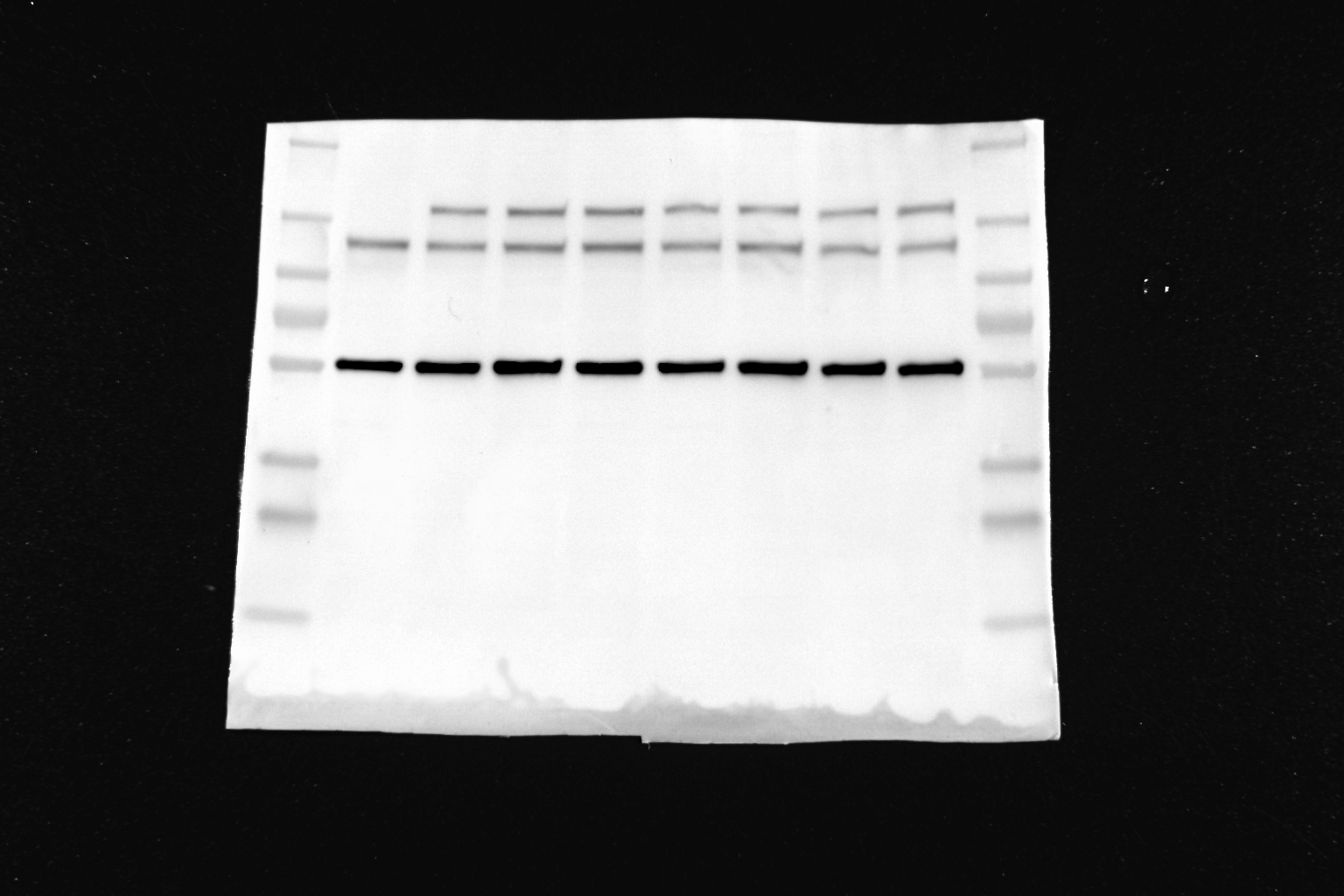

Supplement: Figure 5—figure supplement 1—source data 1. — Raw images and uncropped annotated image of western blots of CLS-2::GFP fusion protein variants in full-protein worm extracts. [file elife-82579-fig5-figsupp1-data1.zip › Figure 5—figure supplement 1—source data 1/WB4_antiCLS-2_antiTub_Composit.tif]

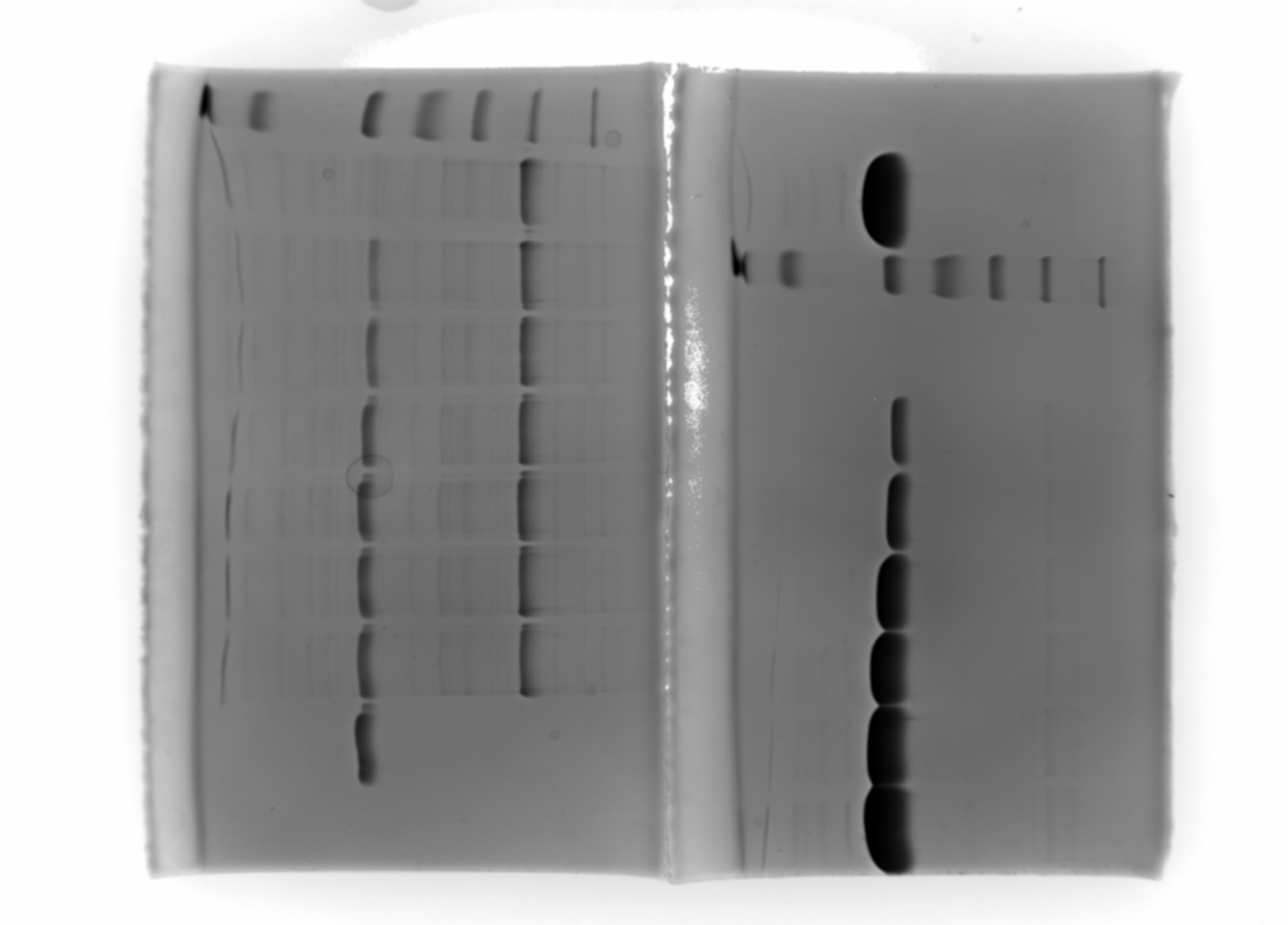

Supplement: Figure 6—figure supplement 1—source data 1. — Raw images and uncropped annotated image of Coomassie-stained gels for purification of BUB-1, HCP-1, CLS-2::GFP and CLS-2R970A::GFP proteins, and of microtubule/BUB-1 pelleting assay. [file elife-82579-fig6-figsupp1-data1.zip › Figure 6—figure supplement 1—source data 1/A_BUB-1_Coomassie_pelleting assay.tif]

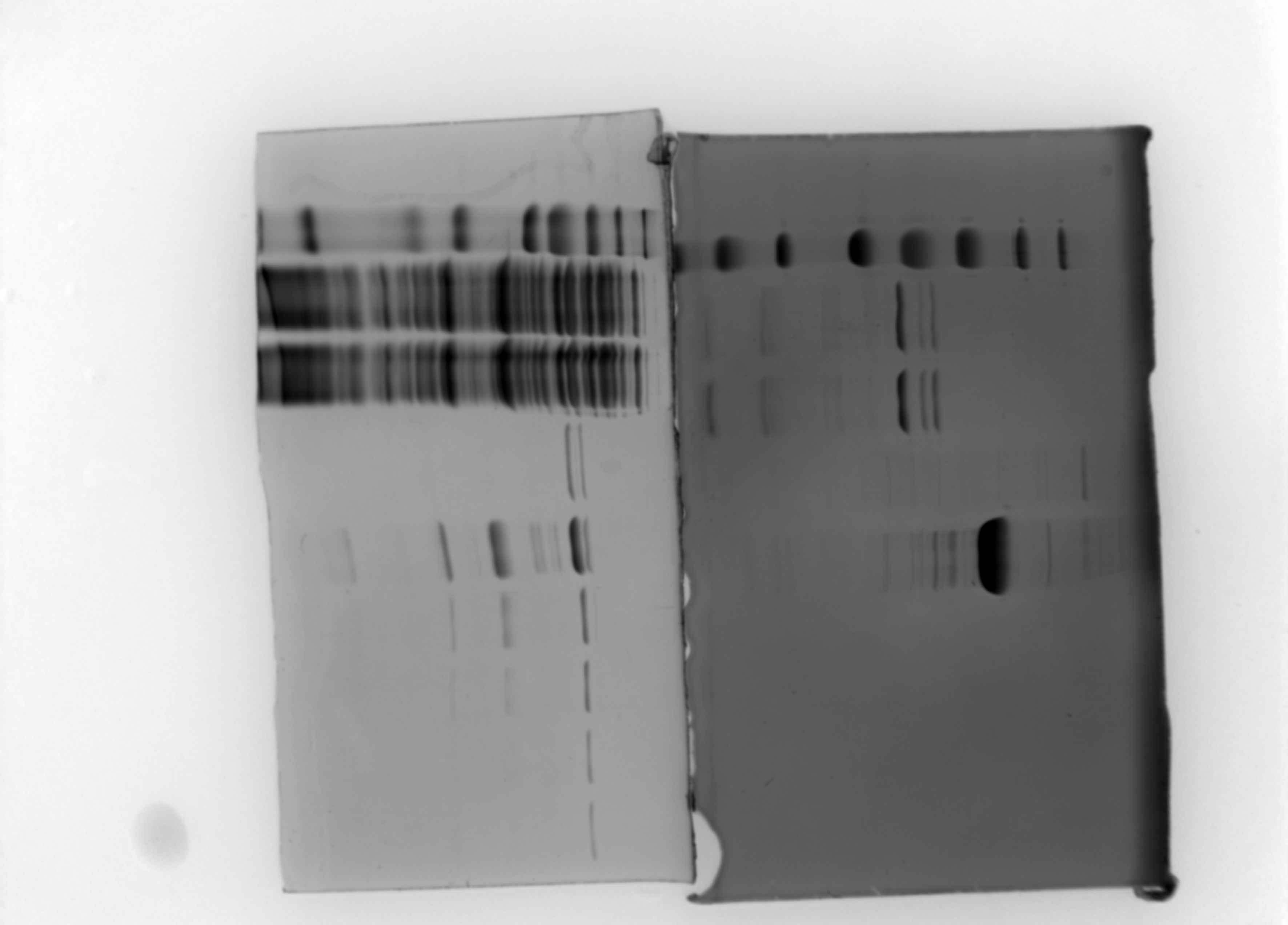

Supplement: Figure 6—figure supplement 1—source data 1. — Raw images and uncropped annotated image of Coomassie-stained gels for purification of BUB-1, HCP-1, CLS-2::GFP and CLS-2R970A::GFP proteins, and of microtubule/BUB-1 pelleting assay. [file elife-82579-fig6-figsupp1-data1.zip › Figure 6—figure supplement 1—source data 1/B_HCP-1_150507_coomassie.tif]

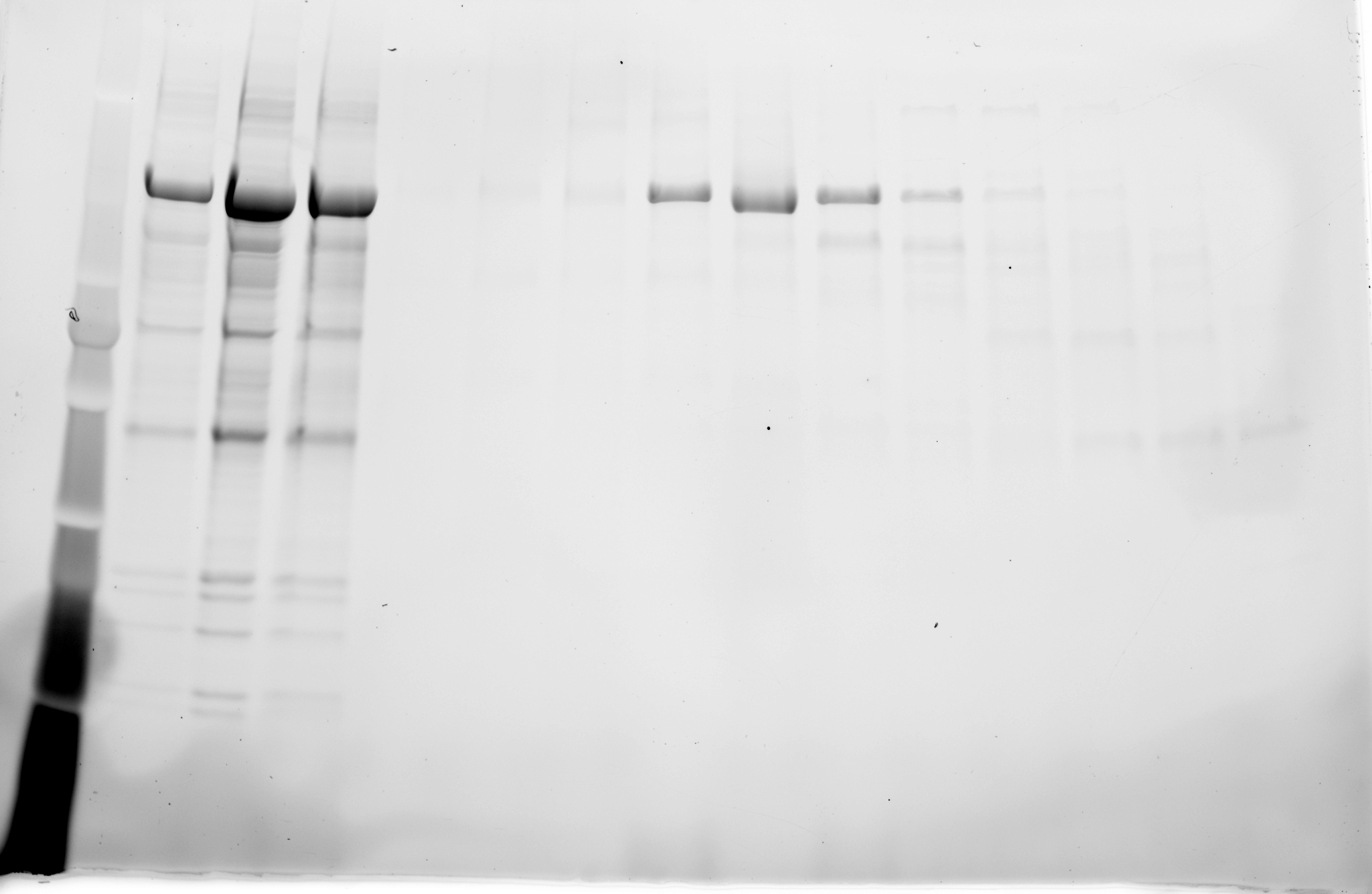

Supplement: Figure 6—figure supplement 1—source data 1. — Raw images and uncropped annotated image of Coomassie-stained gels for purification of BUB-1, HCP-1, CLS-2::GFP and CLS-2R970A::GFP proteins, and of microtubule/BUB-1 pelleting assay. [file elife-82579-fig6-figsupp1-data1.zip › Figure 6—figure supplement 1—source data 1/C_CLS-2_Coomassie_2019-07-31_GF.jpg]

TOGL2

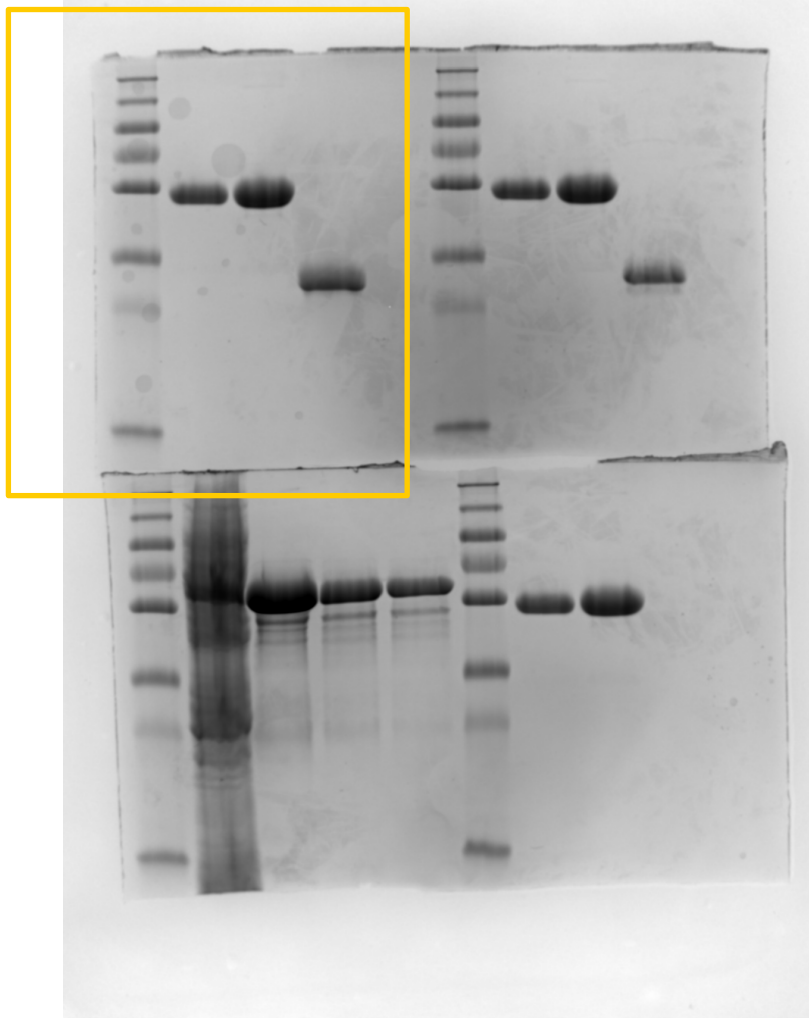

TOGL3

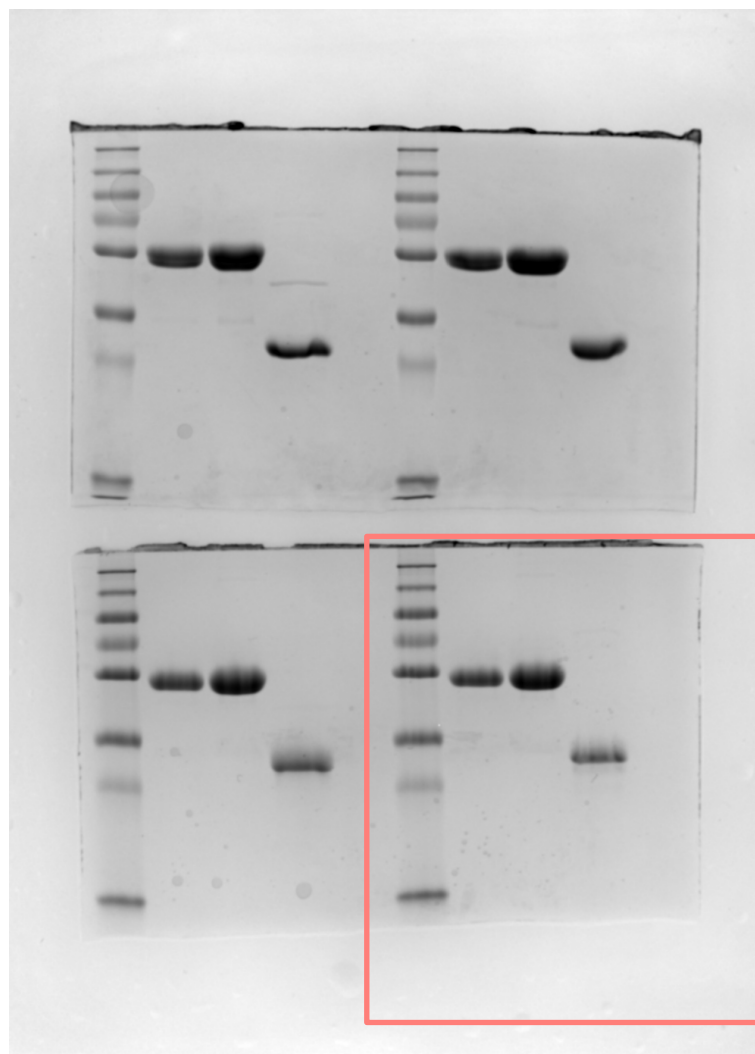

Supplement: Figure 6—figure supplement 1—source data 2. — Raw images and uncropped annotated image of Coomassie-stained gels for protein fractions of gel filtration assay. [file elife-82579-fig6-figsupp1-data2.zip › Figure 6—figure supplement 1—source data 2/GF_TOGL2_TOGL3_annotated.pdf]

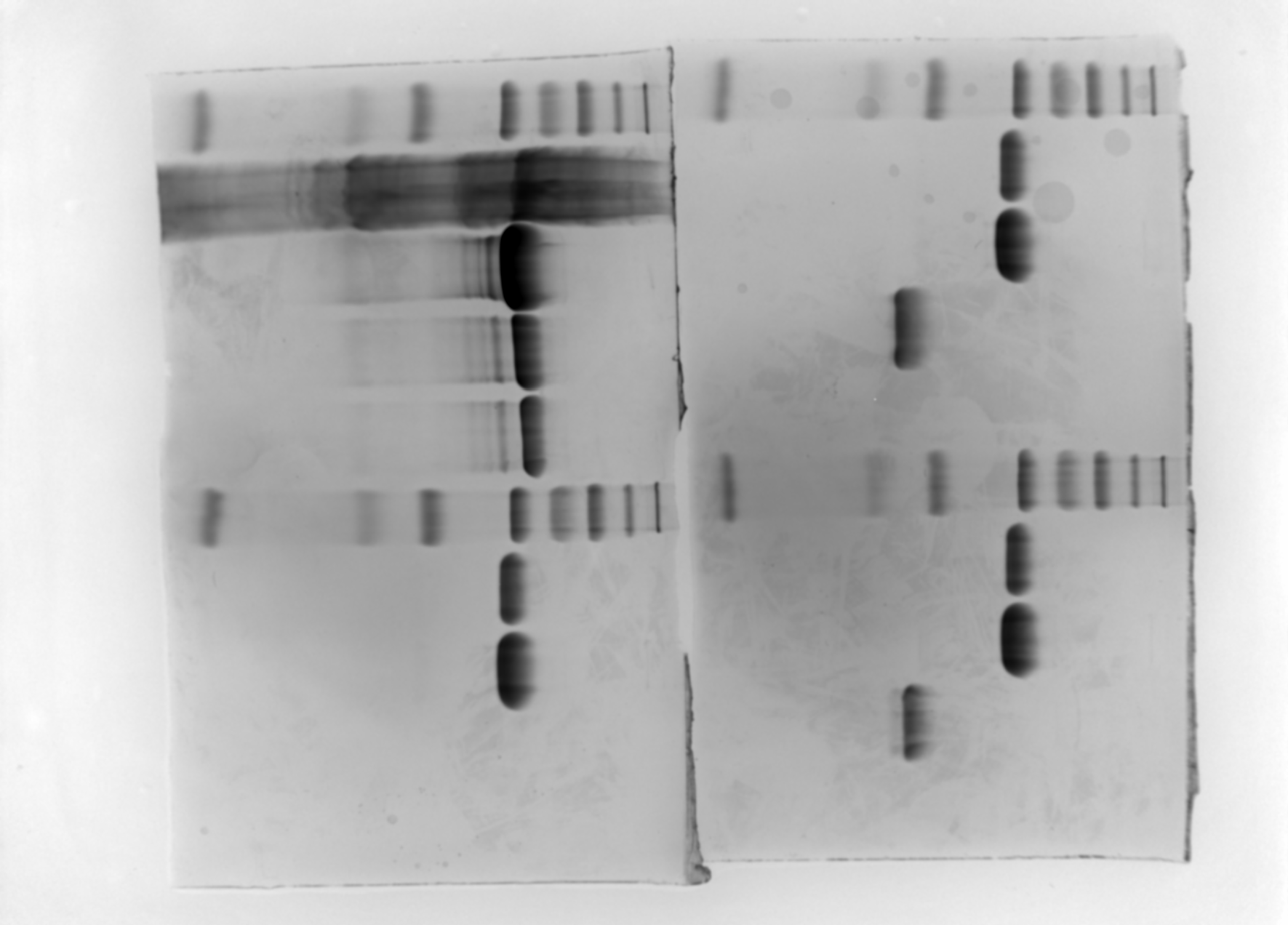

Supplement: Figure 6—figure supplement 1—source data 2. — Raw images and uncropped annotated image of Coomassie-stained gels for protein fractions of gel filtration assay. [file elife-82579-fig6-figsupp1-data2.zip › Figure 6—figure supplement 1—source data 2/GF_TOGL2_top-left_LAS-4000 2018-04-26 18hr 40min.tif]

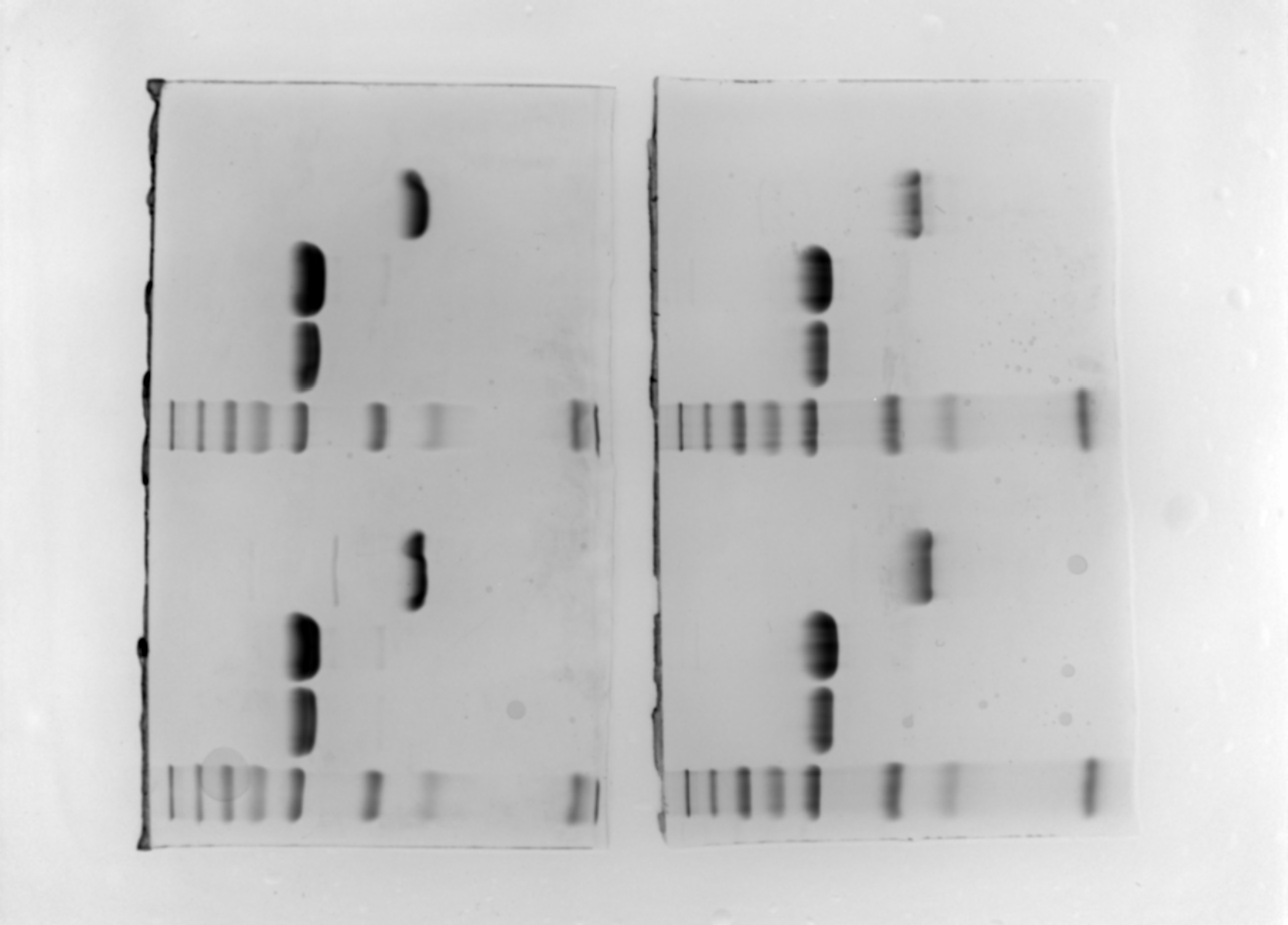

Supplement: Figure 6—figure supplement 1—source data 2. — Raw images and uncropped annotated image of Coomassie-stained gels for protein fractions of gel filtration assay. [file elife-82579-fig6-figsupp1-data2.zip › Figure 6—figure supplement 1—source data 2/GF_TOGL3_bottom-right_LAS-4000-2018-04-21_13hr25min.tif]
