# Supplementary material for: Synergistic stabilization of microtubules by BUB-1, HCP-1, and CLS-2 controls microtubule pausing and meiotic spindle assembly
Source: eLife. 2023 Feb 17;12:e82579. doi: 10.7554/eLife.82579 (PMC10005782; doi:10.7554/eLife.82579)
Supplement: Figure 2—figure supplement 1—source data 2. — Raw images and uncropped annotated images of western blots of GFP::HCP-1 fusion protein variants in full-protein worm extracts. [file elife-82579-fig2-figsupp1-data2.zip › Figure 2—figure supplement 1—source data 2/Figure 2_Figure supplement 1_source data 2_Panel C source data.pdf]

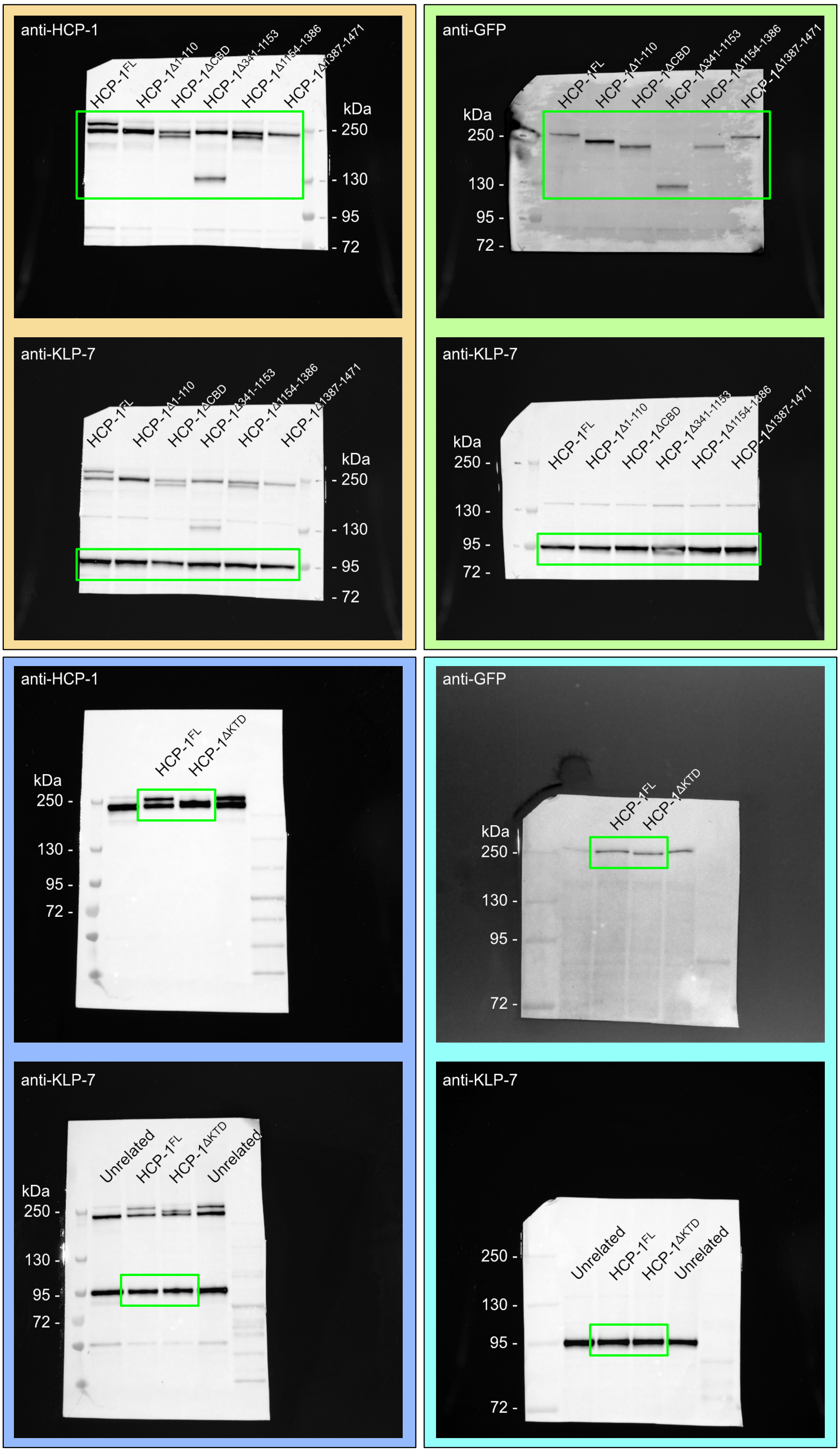

Figure 2 – Figure supplement 1 – source data 2 – Panel C source data

Uncropped images of Western blots of full-protein worm extracts (50 worms per sample) to detect indicated transgenes specifically (anti-GFP) or both the transgene and endogenous HCP-1 (anti-HCP-1), as well as controls (anti-KLP-7). Bands of interest are indicated with a green box. Colored frameworks indicate the same membrane. Molecular weight ladder, Thermofisher prestained PageRuler.
