# Supplementary material for: Synergistic stabilization of microtubules by BUB-1, HCP-1, and CLS-2 controls microtubule pausing and meiotic spindle assembly
Source: eLife. 2023 Feb 17;12:e82579. doi: 10.7554/eLife.82579 (PMC10005782; doi:10.7554/eLife.82579)
Supplement: Figure 5—figure supplement 1—source data 1. — Raw images and uncropped annotated image of western blots of CLS-2::GFP fusion protein variants in full-protein worm extracts. [file elife-82579-fig5-figsupp1-data1.zip › Figure 5—figure supplement 1—source data 1/Figure 5 – Figure supplement 1 – source data 1 - Panel D source data_IMAGES.pdf]

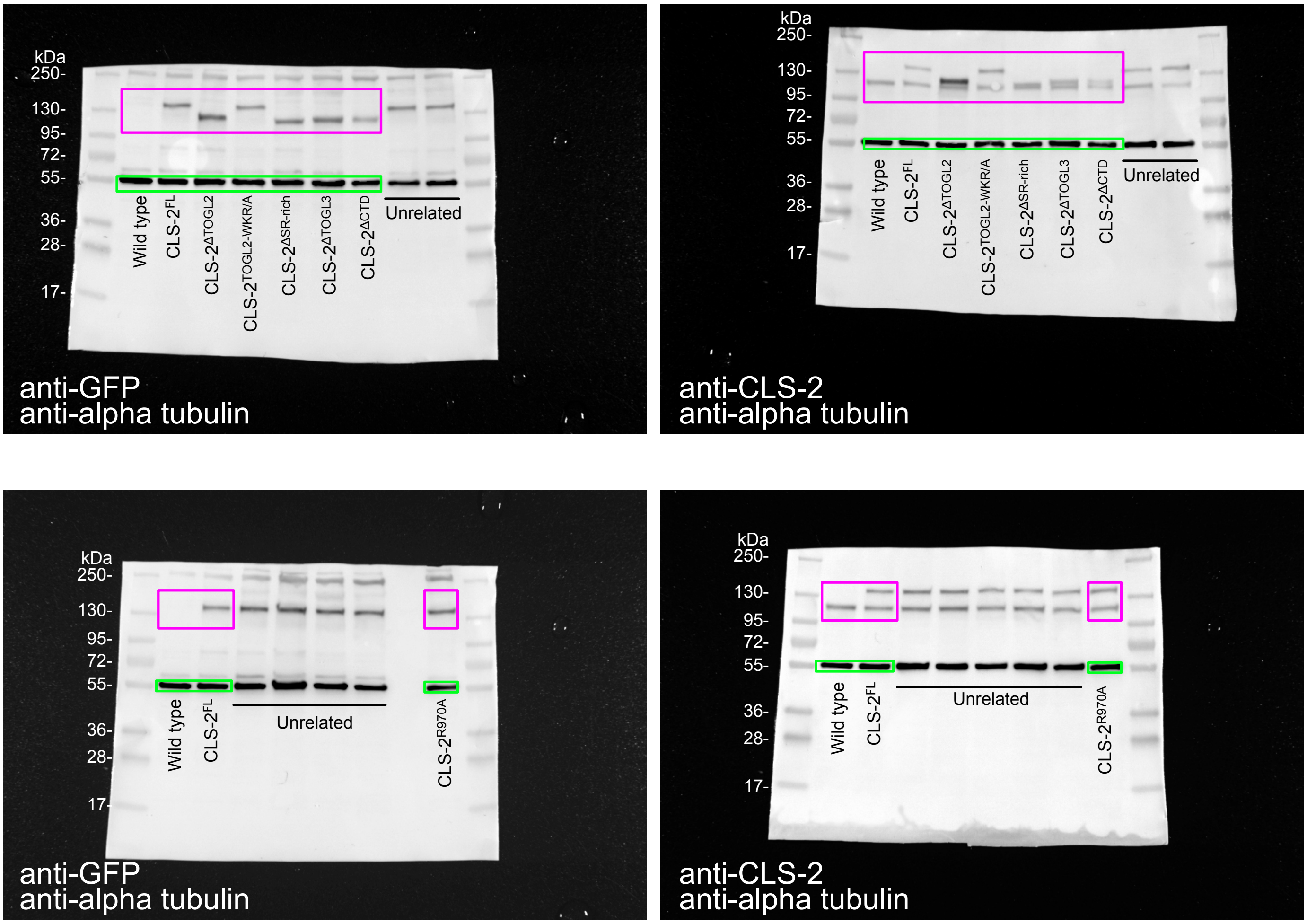

Figure 5 - Figure supplement 1 - source data 1 - Panel D source data. Uncropped images of Western Blots of transgenic worm full-protein extracts (100 worms per sample). Bands of interest are shown in magenta boxes, controls in green boxes. Molecular weight ladder, Thermofischer Prestain PageRuler .
