# Supplementary material for: Synergistic stabilization of microtubules by BUB-1, HCP-1, and CLS-2 controls microtubule pausing and meiotic spindle assembly
Source: eLife. 2023 Feb 17;12:e82579. doi: 10.7554/eLife.82579 (PMC10005782; doi:10.7554/eLife.82579)
Supplement: Figure 6—figure supplement 1—source data 1. — Raw images and uncropped annotated image of Coomassie-stained gels for purification of BUB-1, HCP-1, CLS-2::GFP and CLS-2R970A::GFP proteins, and of microtubule/BUB-1 pelleting assay. [file elife-82579-fig6-figsupp1-data1.zip › Figure 6—figure supplement 1—source data 1/Figure 6_Figure supplement 1_Source data 1_Panel A-B source data.pdf]

A

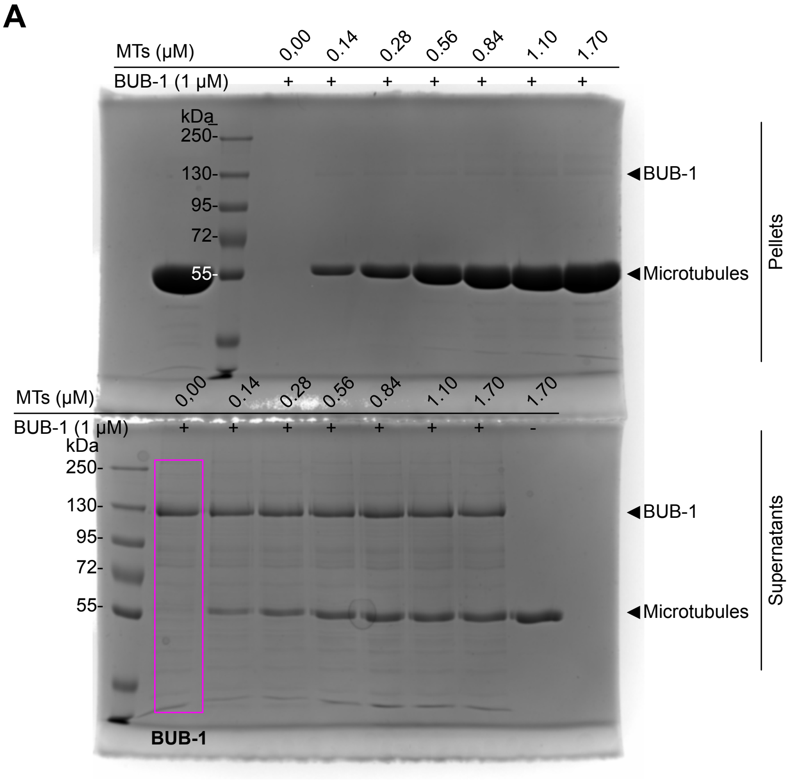

B

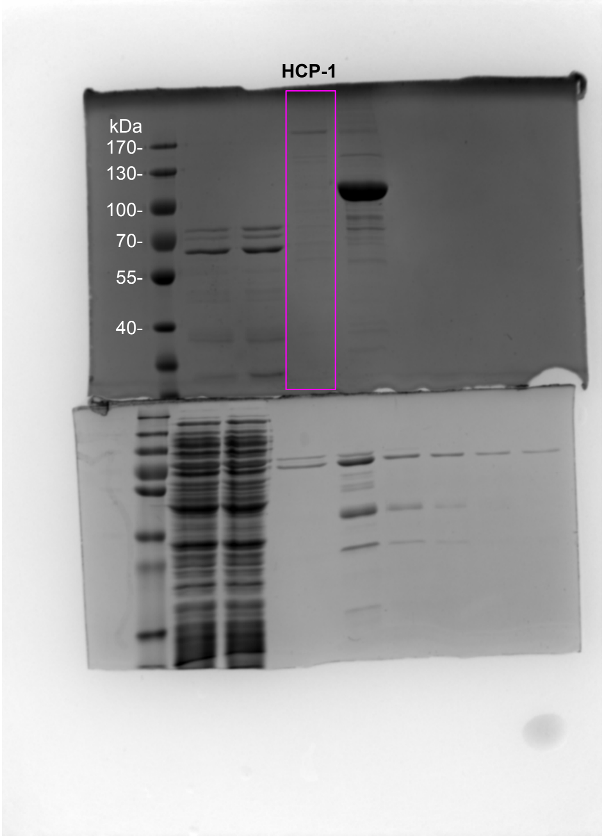

C

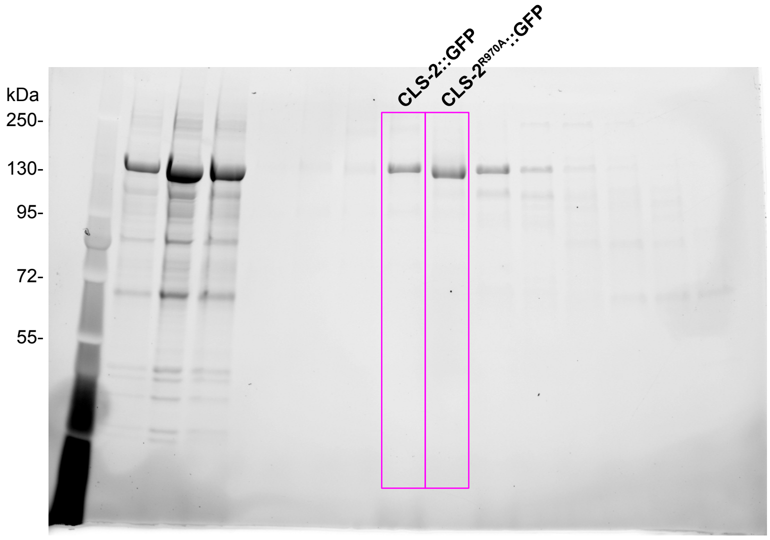

Figure 6 - Figure supplement 1 - Source data 1 - Panel A, B source data.
(A) Uncropped coomassie-stained gel of purified BUB-1 (magenta box), and microtubule pelleting assay. Presence (+) or absence (-) of 1 μM purified BUB-1 is shown, as well as the concentration of microtubules (MTs, μM). (B, C) Uncropped Coomassie-stained gels of purified HCP-1 (B), CLS-2::GFP and CLS-2^{R970A}::GFP (C) (lane of interest in magenta box).Prestained protein size ladders, Thermofisher (A, C) and Euromedex (B).
