# Supplementary material for: Synergistic stabilization of microtubules by BUB-1, HCP-1, and CLS-2 controls microtubule pausing and meiotic spindle assembly
Source: eLife. 2023 Feb 17;12:e82579. doi: 10.7554/eLife.82579 (PMC10005782; doi:10.7554/eLife.82579)
Supplement: MDAR checklist [file elife-82579-mdarchecklist1.pdf]

Westbrook Centre
Milton Road
Cambridge CB4 1YG, UK
P 01223 855340
W elifesciences.org
T @elife

Materials Design Analysis Reporting (MDAR) Checklist for Authors

The MDAR framework establishes a minimum set of requirements in transparent reporting mainly applicable to studies in the life sciences.

eLife asks authors to **provide detailed information within their article** to facilitate the interpretation and replication of their work. Authors can also upload supporting materials to comply with relevant reporting guidelines for health-related research (see EQUATOR Network), life science research (see the BioSharing Information Resource), or animal research (see the ARRIVE Guidelines and the STRANGE Framework; for details, see *eLife*'s Journal Policies). Where applicable, authors should refer to any relevant reporting standards materials in this form.

For all that apply, please note **where in the article** the information is provided. Please note that we also collect information about data availability and ethics in the submission form.

Materials:

Newly created materials	Indicate where provided: section/figure legend	N/A
The manuscript includes a dedicated "materials availability statement" providing transparent disclosure about availability of newly created materials including details on how materials can be accessed and describing any restrictions on access.	All *C. elegans* strains produced for this study are listed in [Supplementary Material - Table S1]. They will be made available upon request as stated in section: Methods/*C. elegans* strain maintenance.<br>Microscope acquisitions cannot share directly due to their size. However, they will be made available without restriction upon request to a corresponding author. Numerical data of microscope sample analyses were extracted using Fiji which is freely available on all platforms at https://imagej.net/software/fiji/downloads .<br>They are provided in source data.	

Antibodies	Indicate where provided: section/figure legend	N/A
For commercial reagents, provide supplier name, catalogue number and RRID, if available.	Details on antibodies used are provided in following paragraphs of the of Methods section: Live Imaging and image analysis; Western blotting.	

DNA and RNA sequences	Indicate where provided: section/figure legend	N/A
Short novel DNA or RNA including primers, probes: Sequences should be included or deposited in a public repository.	Primer sequences for double-stranded RNA production are provided in Methods/ RNA-mediated interference.	

Cell materials	Indicate where provided: section/figure legend	N/A
Cell lines: Provide species information, strain. Provide accession number in repository OR supplier name, catalog number, clone number, OR RRID.	Yeast strains used for yeast two hybrid are provided in Methods/ Yeast two hybrid assay.<br>Cell lines used for protein purification are provided in Methods/ Protein production and purification.	
Primary cultures: Provide species, strain, sex of origin, genetic modification status.		N/A

Experimental animals	Indicate where provided: section/figure legend	N/A
Laboratory animals or Model organisms: Provide species, strain, sex, age, genetic modification status. Provide accession number in repository OR supplier name, catalog number, clone number, OR RRID.	Details on *C. elegans* strains used are available in Methods/ *C. elegans* strain maintenance, and in<br>[Supplementary Material - Table S1].	
Animal observed in or captured from the field: Provide species, sex, and age where possible.		N/A

Plants and microbes	Indicate where provided: section/figure legend	N/A
Plants: provide species and strain, ecotype and cultivar where relevant, unique accession number if available, and source (including location for collected wild specimens).		N/A
Microbes: provide species and strain, unique accession number if available, and source.		N/A

**Human research participants**	**Indicate where provided: section/figure legend) or state if these demographics were not collected**	**N/A**
If collected and within the bounds of privacy constraints report on age, sex, gender and ethnicity for all study participants.		N/A

Design:

**Study protocol**	**Indicate where provided: section/figure legend**	**N/A**
If the study protocol has been pre-registered, provide DOI. For clinical trials, provide the trial registration number OR cite DOI.		N/A

**Laboratory protocol**	**Indicate where provided: section/figure legend**	**N/A**
Provide DOI OR other citation details if detailed step-by-step protocols are available.		N/A

**Experimental study design (statistics details) ***		
**For in vivo studies: State whether and how the following have been done**	**Indicate where provided: section/figure legend. If it could have been done, but was not, write "not done"**	**N/A**
Sample size determination	For protein localization, sample sizes are shown in figure legends.<br>For viability assays, sample sizes are shown in respective source data files.<br>For live imaging experiments, sample sizes can be found in corresponding figure panels.<br>For Western blots of worm full extracts, samples sizes are indicated in respective figure legends.<br>For all figures, sample sizes were determined based on previously published datasets as well as on practical constrains imposed by the method applied.	
Randomisation		N/A
Blinding	Worms were only selected for their development stage (L4) at the beginning of the experiment,	

	corresponding to the proper stage for RNAi treatment. No further selection was done.	
Inclusion/exclusion criteria		N/A

**Sample definition and in-laboratory replication**	**Indicate where provided: section/figure legend**	**N/A**
State number of times the experiment was replicated in the laboratory.	Live imaging of oocytes and one-cell embryos were performed at least twice per genotype, at least one week apart (i.e. at least over 3 generations).<br>Yeast two hybrid assay was performed twice independently.<br>For in vitro microtubule dynamics, a minimum of 3 independent experiments were made per condition.	
Define whether data describe technical or biological replicates.	Biological replicates:<br>For immunolocalization experiments, a maximum of two cells were captured per worm.<br>For Live imaging, each imaged cell comes from an unique worm.<br>For Y2H, yeasts were transformed and mated twice.<br>Technical replicates:<br>For in vitro microtubule dynamics and assays, independent experiments were performed using the same batch of purified proteins.	

**Ethics**	**Indicate where provided: section/submission form**	**N/A**
Studies involving human participants: State details of authority granting ethics approval (IRB or equivalent committee(s), provide reference number for approval.		N/A
Studies involving experimental animals: State details of authority granting ethics approval (IRB or equivalent committee(s), provide reference number for approval.		N/A
Studies involving specimen and field samples: State if relevant permits obtained, provide details of authority approving study; if none were required, explain why.		N/A

Dual Use Research of Concern (DURC)	Indicate where provided: section/submission form	N/A
If study is subject to dual use research of concern regulations, state the authority granting approval and reference number for the regulatory approval.		N/A

Analysis:

Attrition	Indicate where provided: section/figure legend	N/A
Describe whether exclusion criteria were pre-established. Report if sample or data points were omitted from analysis. If yes, report if this was due to attrition or intentional exclusion and provide justification.		N/A

Statistics	Indicate where provided: section/figure legend	N/A
Describe statistical tests used and justify choice of tests.	- **Dataset and statistics for figure 1B** is provided in file [Figure 1 – source data 1 - Panel B source data]. Mean of BUB-1 fluorescence signal intensity/Noise at kinetochores in *bub-3Δ* was compared to that of N2 control using a two-tailed t-test to determine whether there is a difference between the two genotypes.<br>- **Datasets and statistics for Figure 1 - Figure supplement 1 panel B** are provided in [Figure 1 - Figure supplement 1 - source data 3 - Panel B source data]. Mean of CLS-2 fluorescence signal intensity/Noise at kinetochores in *bub-3Δ* was compared to that of N2 control using a two-tailed t-test to determine whether there is a difference between the two genotypes.<br>**- Datasets and statistics for Figure 1 - Figure supplement 1 panel C** are provided in [Figure 1 - Figure supplement 1 - source data 4 - Panel C source data]. Mean of HCP-1 fluorescence signal intensity/Noise of the whole spindle in *bub-3Δ* mutants (JDU755) was compared to that of controls (PHX441) using a two-tailed t-test to determine whether there is a difference between the	

	two genotypes.<br>- **Datasets and multiple comparison statistics for figures 3B and 4C** are reported in [Figure 3 – source data 2 – Panel B source data] and [Figure 4 – source data 2 – Panel C source data], respectively. For both, each condition was tested for normal distribution using Anderson-Darling, D'Agostino and Pearson, Shapiro-Wilk and Kolmogorov-Smirnov tests. If all tests passed, ANOVA was used to compare RNAi treated samples to No RNAi control. If normality was not checked, or if a sample was small, a non-parametric Kruskal-Wallis test was used.<br>- **Dataset and statistics for Figure 3_Figure supplement 1 panel C** are reported in [Figure 3 – Figure supplement 1 - source data 2 – Panel B-C source data]. Non-parametric tests (Kruskal-Wallis for multiple comparisons or Mann-Whitney for pairwise comparisons) because N for some samples was small.<br>- Raw data for **figure 6C to F** are reported in [Figure 6 – source data 1 – Panel B-F source data_BL], analyzed datasets and statistics are reported in [Figure 6 – source data 2 - B-F statistics source data]. For figure 6C, ANOVA multiple comparisons were performed using Tukey-Kramer test to compare sample means allowing the possibility of unequal sample sizes. Results for comparisons to the control are reported on the graph. For figures 6D-F, ANOVA multiple comparisons were performed using Dunnett test to compare samples to one reference. A first test was performed to compare all samples to the control. Then, a second test was run in order to compare the effect of adding CLS-2^{R970A} or multiple proteins (including CLS-2) to CLS-2 alone. All statistical tests were performed on data calculated per microtubule. For panel C, dot plots are direct representation of data. For panels D-F, histograms represent the average number of events.	

Data availability	Indicate where provided: section/submission form	N/A
For newly created and reused datasets, the manuscript includes a data availability statement that provides details for access (or notes restrictions on access).	All newly created datasets are available in source data without any restriction on access. No dataset was reused.	
When newly created datasets are publicly available, provide accession number in repository OR DOI and licensing details where available.		N/A
If reused data is publicly available provide accession number in repository OR DOI, OR URL, OR citation.		N/A

Code availability	Indicate where provided: section/figure legend	N/A
For any computer code/software/mathematical algorithms essential for replicating the main findings of the study, whether newly generated or re-used, the manuscript includes a data availability statement that provides details for access or notes restrictions.		N/A
Where newly generated code is publicly available, provide accession number in repository, OR DOI OR URL and licensing details where available. State any restrictions on code availability or accessibility.		N/A
If reused code is publicly available provide accession number in repository OR DOI OR URL, OR citation.		N/A

Reporting:

The MDAR framework recommends adoption of discipline-specific guidelines, established and endorsed through community initiatives.

Adherence to community standards	Indicate where provided: section/figure legend	N/A
State if relevant guidelines (e.g., ICMJE, MIBBI, ARRIVE, STRANGE) have been followed, and whether a checklist (e.g., CONSORT, PRISMA, ARRIVE) is provided with the manuscript.		

---

* We provide the following guidance regarding transparent reporting and statistics; we also refer authors to

Ten common statistical mistakes to watch out for when writing or reviewing a manuscript.

Sample-size estimation

- You should state whether an appropriate sample size was computed when the study was being designed
- You should state the statistical method of sample size computation and any required assumptions
- If no explicit power analysis was used, you should describe how you decided what sample (replicate) size (number) to use

Replicates

- You should report how often each experiment was performed
- You should include a definition of biological versus technical replication
- The data obtained should be provided and sufficient information should be provided to indicate the number of independent biological and/or technical replicates
- If you encountered any outliers, you should describe how these were handled
- Criteria for exclusion/inclusion of data should be clearly stated
- High-throughput sequence data should be uploaded before submission, with a private link for reviewers provided (these are available from both GEO and ArrayExpress)

Statistical reporting

- Statistical analysis methods should be described and justified
- Raw data should be presented in figures whenever informative to do so (typically when N per group is less than 10)
- For each experiment, you should identify the statistical tests used, exact values of N, definitions of center, methods of multiple test correction, and dispersion and precision measures (e.g., mean, median, SD, SEM, confidence intervals; and, for the major substantive results, a measure of effect size (e.g., Pearson's r, Cohen's d)
- Report exact p-values wherever possible alongside the summary statistics and 95% confidence intervals. These should be reported for all key questions and not only when the p-value is less than 0.05.

Group allocation

- Indicate how samples were allocated into experimental groups (in the case of clinical studies, please specify allocation to treatment method); if randomization was used, please also state if restricted randomization was applied
- Indicate if masking was used during group allocation, data collection and/or data analysis
