# Supplementary material for: Synergistic stabilization of microtubules by BUB-1, HCP-1, and CLS-2 controls microtubule pausing and meiotic spindle assembly
Source: eLife. 2023 Feb 17;12:e82579. doi: 10.7554/eLife.82579 (PMC10005782; doi:10.7554/eLife.82579)
Supplement: Supplementary file 1. [file elife-82579-supp1.docx]

Supplementary File 1 List of *C. elegans* strains.

**Strain Code Genotype**

JDU13 ijmSi4[Pmex-5::cls-2ΔSR-rich_reencodedreenc::gfp::tbb-2 3' UTR; Cb-unc-119(+)]I; unc-119(ed3)III?.

JDU15 ijmSi5[Pmex-5_cls-2_reencoded_∆TOGL2::gfp::tbb-2 3' UTR; Cb-unc-119(+)]I; unc-119(ed3)III.

JDU22 ijmSi9[Pmex-5::cls-2_reencoded_ΔTOGL3::gfp::tbb-2 3' UTR; Cb-unc-119(+)]I; unc-119(ed3)III?.

JDU26 ijmSi3[Pmex-5::cls-2_reencoded::gfp::tbb-2 3' UTR; Cb-unc-119(+)]I; unc-119(ed3)III?.

JDU28 ltSi259[Phcp-1::GFP::hcp-1_reencoded; Cb-unc-119(+)]I; ltSi44[Pknl-1::knl-1_reencoded_∆85-505)::mCherry; Cb-unc-119(+)]II; unc-119(ed3)III.

JDU31 ijmSi3[Pmex-5::cls-2_reencoded::gfp::tbb-2 3’ UTR; cb-unc-119(+)]I; ltSi44[Pknl-1::knl-1_reencoded_∆85-505)::mCherry; Cb-unc-119(+)]II; unc-119(ed3)III.

JDU35 ijmSi3[Pmex-5::cls-2_reencoded::gfp::tbb-2 3’ UTR; Cb-unc-119(+)]I; ltSi1[Pknl-1::knl-1_reencoded::mCherry; Cb-unc-119(+)]II; unc-119(ed3) III.

JDU36 ltSi259[Phcp-1::gfp::hcp-1_reencoded; Cb-unc-119(+)]I; ltSi1[Pknl-1::knl-1_reencoded::mCherry; Cb-unc-119(+)]II; unc-119(ed3)III.

JDU43 ijmSi15[Pmex-5::cls-2_reencoded_∆Linker::gfp::tbb-2 3' UTR; Cb-unc-119(+)]I; unc-119(ed3)III.

JDU44 ijmSi16[Pmex-5_cls-2_reencoded_ΔTail::gfp::tbb-2 3' UTR; Cb-unc-119(+)]I; unc-119(ed3)III.

JDU69 ijmSi13[Pmex-5::bub-1::sfgfp; Cb-unc-119(+)]I; ltSi1[Pknl-1::knl-1_reencoded::mCherry; Cb-unc-119(+)]II; unc-119(ed3) III.

JDU71 ijmSi13[Pmex-5_bub-1::sfgfp; Cb-unc-119(+)]I; ltSi44[Pknl-1::knl-1_reencoded_∆85-505)::mCherry; Cb-unc-119(+)]II; unc-119(ed3) III.

JDU107 ijmSi3[Pmex-5::cls-2_reencoded::gfp::tbb-2 3' UTR; Cb-unc-119(+)]I; ltSi264[Pbub-1::bub-1_reencoded::mCherry; Cb-unc-119(+)]II; unc-119(ed3)III.

JDU114 ijmSi12[Pmex-5::cls-2_reencoded_∆CTD::gfp::tbb-2 3' UTR; Cb-unc-119(+)]I; unc-119(ed3)III.

JDU132 ijmSi33[Pmex-5::cls-2_reencoded_ΔAB::gfp::tbb-2 3’ UTR; Cb-unc-119(+)]I; unc-119(ed3)III.

JDU134 ijmSi34[Pmex-5::cls-2_reencoded_ΔDE::gfp::tbb-2 3’ UTR; Cb-unc-119(+)]I; unc-119(ed3)III.

JDU135 ijmSi19[Pmex-5::cls-2_reencoded_R970A::GFP tbb-2 3’ UTR; Cb-unc-119(+)]I; unc-119(ed3)III?.

JDU142 ijmSi36[Pmex-5::cls-2::reencoded_ΔAC::gfp::tbb-2 3’ UTR; Cb-unc-119(+)]I; unc-119(ed3)III.

JDU143 ijmSi37[Pmex-5::cls-2::reencoded_ΔBC::gfp::tbb-2 3’ UTR; Cb-unc-119(+)]I; unc-119(ed3)III.

JDU145 ijmSi38[Pmex-5::cls-2::reencoded_ΔBD::gfp::tbb-2 3’ UTR; Cb-unc-119(+)]I; unc-119(ed3)III.

JDU146 ijmSi3[Pmex-5::cls-2_reencoded::gfp::tbb-2 3' UTR; Cb-unc-119 (+)]I; ijmSi31[Pmex-5::mCherry::his-11::tbb-2 3' UTR]II; unc-119(ed3)III.

JDU148 ijmSi39[Pmex-5::cls-2::reencoded_ΔCD::gfp::tbb-2 3’ UTR; Cb-unc-119(+)]I; unc-119(ed3)III.

JDU155 ijmSi4[Pmex-5::cls-2ΔSR-rich_reencoded::gfp::tbb-2 3' UTR; Cb-unc-119 (+)]I; ijmSi31[Pmex-5::mCherry::his-11::tbb-2 3' UTR]II; unc-119(ed3)III.

JDU162 ijmSi12 [Pmex-5::cls-2_reencoded_ΔCTD::gfp::tbb-2 3' UTR; Cb-unc-119 (+)] I; ijmSi31[Pmex-5::mCherry::his-11::tbb-2 3' UTR]II; unc-119(ed3)III.

JDU172 ijmSi5[Pmex-5::cls-2_reencoded_ΔTOGL2::gfp::tbb-2 3' UTR; Cb-unc-119 (+)]I; ijmSi31[Pmex-5::mCherry::his-11::tbb-2 3' UTR]II; unc-119(ed3)III.

JDU187 ijmSi50[Pmex-5::cls-2::reencoded_ΔCE::gfp::tbb-2 3’ UTR; Cb-unc-119(+)]I; unc-119(ed3)III.

JDU168 ijmSi19[Pmex-5::cls-2reencoded_R970A::gfp::tbb-2; Cb-unc-119 (+)]I; ijmSi31[Pmex-5::mCherry::his11::tbb-2 3’ UTR]II; unc-119(ed3)III.

JDU193 ijmSi53[Pmex-5::cls-2::reencoded_W57A_K177A_R224A::gfp::tbb-2 3' UTR]I; unc-119(ed3)III.

JDU205 ijmSi53[Pmex-5::cls-2_reencoded_W57A_K177A_R224A::gfp::tbb-2 3' UTR; Cb-unc-119(+)]I; ijmSi31[Pmex-5::mCherry::his-11::tbb-2 3' UTR]II; unc-119(ed3)III? .

JDU232 ijmSi9[Pmex-5::cls-2_reencoded_ΔTOGL3::gfp::tbb-2 3' UTR; Cb-unc-119(+)]I; ijmSi31[Pmex-5::mCherry::his-11::tbb-2 3' UTR]II; unc-119(ed3)III?.

JDU233 ijmSi63[Pmex-5::gfp::tba-2; mCherry::his-11; Cb-unc-119(+)] II; unc-119(ed3)III?.

JDU243 ijmSi3[Pmex-5::cls-2_reencoded::gfp::tbb-2 3' UTR; Cb-unc-119(+)]I; ijmSi63[Pmex-5::gfp::tba-2; mCherry::his-11; Cb-unc-119(+)]II; unc-119(ed3)III?.

JDU244 ltSi259[Phcp-1::gfp::hcp-1_reencoded; Cb-unc-119(+)]I; ijmSi31[Pmex-5::mCherry::his11::tbb-2 3’ UTR]II; unc-119(ed3)III.

JDU255 ijmSi98[Pmex-5::mCherry::his11::tbb-2 3' UTR]II; unc-119(ed3)III?; hcp-2(ijm6)V, ddIs6[gfp::tbg-1; unc-119(+)]V.

JDU259 ijmSi68[Phcp-1::gfp::hcp-1_reencoded_∆220-1153; Cb-unc-119(+)]I; ijmSi69[Pmex-5::cls-2::reencoded::mCherry::tbb-2 3' UTR]II; unc-119(ed3)III?.

JDU263 ijmSi19[Pmex-5::cls-2_reencoded_R970A::gfp::tbb-2; Cb-unc-119(+)]I; ijmSi63[Pmex-5::gfp::tba-2; mCherry::his-11; Cb-unc-119(+)]II; unc-119(ed3)III?.

JDU264 ijmSi12[Pmex-5::cls-2_reencoded_∆CTD::gfp::tbb-2 3’ UTR; Cb-unc-119(+)]I; ijmSi63[pJD520; Pmex-5::GFP::tba-2; mCherry::his-11; Cb-unc-119(+)]II; unc-119(ed3)III?.

JDU266 ijmSi7[Pmex-5::gfp::tbb-2; mCherry::his-11; Cb-unc-119(+)]I; ltSi264[Pbub-1::bub-1_reencoded::mCherry; Cb-unc-119(+)]II; unc-119(ed3)III?.

JDU269 ltSi259[Phcp-1::gfp::hcp-1 _reencoded; Cb-unc-119(+)]I; ijmSi69[Pmex-5::cls-2_reencoded::mCherry::tbb-2 3' UTR]II; unc-119(ed3)III?.

JDU270 ijmSi58[Phcp-1::gfp::hcp-1_reencoded_∆1-219; Cb-unc-119(+)]I; ijmSi69[Pmex-5::cls-2_reencoded::mCherry::tbb-2 3' UTR]II; unc-119(ed3)III?.

JDU282 ijmSi53[Pmex-5::cls-2_reencoded_W57A_K177A_R224A::gfp::tbb-2 3' UTR; Cb-unc-119(+)]I; ijmSi63[Pmex-5::gfp::tba-2; mCherry::his-11; Cb-unc-119(+)]II; unc-119(ed3)III?.

JDU302 unc-119(ed3)III; hcp-2(ijm6)V; unc-119(+)?.

JDU321 ijmSi7[Pknl-1::knl-1_reencoded::mCherry; Cb-unc-119(+)]II; unc-119(ed3)III?.

JDU323 ijmSi7 [Pknl-1::knl-1_reencoded_∆85-505::mCherry; Cb-unc-119(+)]II; unc-119(ed3)III?.

JDU326 ltSi259[Phcp-1::gfp::hcp-1_reencoded; Cb-unc-119(+)]I; ltSi264[Pbub-1::bub-1_reencoded::mCherry; Cb-unc-119(+)]II; unc-119(ed3)III?.

JDU329 ltSi259[Phcp-1::gfp::hcp-1_reencoded; Cb-unc-119(+)]I; ltSi758[Pbub-1::bub-1_reencoded_D814N::mCherry; Cb-unc-119(+)]II; unc-119(ed3)III?.

JDU333 ijmSi73[Phcp-1::gfp::hcp-1_reencoded_∆1387-1440]I; ijmSi69[Pmex-5::cls-2_reencoded::mCherry::tbb-2 3’ UTR]II; unc-119(ed3)III?.

JDU335 ijmSi76[Phcp-1::gfp::hcp-1_reencoded_∆111-219; Cb-unc-119(+)]I; ijmSi69[Pmex-5::cls-2_reencoded::mCherry::tbb-2 3' UTR]II; unc-119(ed3)III?.

JDU336 ijmSi4[Pmex-5::cls-2_reencoded_ΔSR-rich::gfp::tbb-2 3' UTR; Cb-unc-119(+)]I; ijmSi63[Pmex-5::gfp::tba-2; mCherry::his-11; Cb-unc-119(+)]II; unc-119(ed3)III?.

JDU338 ijmSi80[Phcp-1::gfp::hcp-1_reencoded_∆1154-1386; Cb-unc-119(+)]I; ijmSi69[Pmex-5::cls-2::reencoded::mCherry::tbb-2 3' UTR]II; unc-119(ed3)III?.

JDU351 ijmSi81[Phcp-1::gfp::hcp-1_reencoded_∆1387-1471; Cb-unc-119(+)]I; ijmSi69[Pmex-5_cls-2_reencoded::mCherry::tbb-2 3’ UTR]II.

JDU369 ijmSi83[Phcp-1::gfp::hcp-1_reencoded_∆452-1153; Cb-unc-119(+)]I; ijmSi69[Pmex-5::cls-2_reencoded::mCherry::tbb-2 3' UTR]II; unc-119(ed3)III?.

JDU370 ijmSi84[Phcp-1::gfp::hcp-1_reencoded_∆1-110; Cb-unc-119(+)]; ijmSi69[Pmex-5::cls-2_reencoded::mCherry::tbb-2 3' UTR]II; unc-119(ed3)III?.

JDU373 ijmSi86[Phcp-1::hcp-1_reencoded_∆1472-1475::gfp; Cb-unc-119(+)]I.

JDU374 ijmSi87[Phcp-1::hcp-1_reencoded::gfp; Cb-unc-119(+)]I.

JDU377 ijmSi88[Phcp-1::gfp::hcp-1_reencoded_∆1154-1230; Cb-unc-119(+)]I; unc-119(ed3)III.

JDU378 ijmSi89[Phcp-1::gfp::hcp-1_reencoded_∆1154-1310; Cb-unc-119(+)]I; unc-119(ed3)III.

JDU402 ijmSi90[Phcp-1::gfp::hcp-1_reencoded_∆1-12; Cb-unc-119(+)]I;ijmSi69[Pmex-5::cls-2_reencoded::mCherry::tbb-2 3' UTR]II; unc-119(ed3)III?.

JDU403 ijmSi91[Phcp-1::gfp::hcp-1_reencoded_∆220-340; Cb-unc-119(+)]I; ijmSi69[Pmex-5::cls-2_reencoded::mCherry::tbb-2 3' UTR]II; unc-119(ed3)III.

JDU411 ltSi259[Phcp-1::gfp::hcp-1_reencoded; Cb-unc-119(+)]I; ijmSi31[Pmex-5::mCherry::his-11::tbb-2 3' UTR]II; unc-119(ed3)III?; hcp-2(ijm6)V, ddIs6[gfp::tbg-1; unc-119(+)]V.

JDU414 ijmSi84[Phcp-1::gfp::hcp-1_reencoded_∆1-110; Cb-unc-119(+)]I; ijmSi31[Pmex-5::mCherry::his11::tbb-2 3’ UTR]II; unc-119(ed3) III?; hcp-2(ijm6)V, ddIs6[gfp::tbg-1; unc-119(+)]V.

JDU416 ijmSi92[Phcp-1::gfp::hcp-1_reencoded_∆341-451; Cb-unc-119(+)]I; ijmSi69[Pmex-5::cls-2_reencoded::mCherry::tbb-2 3' UTR]II; unc-119(ed3)III.

JDU423 ijmSi93[Phcp-1::gfp::hcp-1_reencoded_∆46-110; Cb-unc-119(+)]I; ijmSi69[Pmex-5::cls-2_reencoded::mCherry::tbb-2 3' UTR]II; unc-119(ed3)III?.

JDU429 ijmSi81[Phcp-1::gfp::hcp-1_reencoded_∆1387-1471; Cb-unc-119(+)]I; ijmSi31[Pmex-5::mCherry::his11::tbb-2 3' UTR]II; unc-119(ed3)III?; hcp-2(ijm6)V, ddIs6[gfp::tbg-1; unc-119(+)]V.

JDU430 ijmSi80[Phcp-1::gfp::hcp-1_reencoded_Δ1154-1386; Cb-unc-119(+)]I; ijmSi31[Pmex-5::mCherry::his-11::tbb-2 3' UTR]II; unc-119(ed3)III?; hcp-2(ijm6)V, ddIs6[gfp::tbg-1; unc-119(+)]V.

JDU434 ijmSi94[Phcp-1::gfp::hcp-1_reencoded_Δ111-340; Cb-unc-119(+)]I; ijmSi69[Pmex-5::cls-2_reencoded::mCherry::tbb-2 3' UTR]II; unc-119(ed3)III?.

JDU435 ijmSi95[Phcp-1::gfp::hcp-1_reencoded_∆341-1153; Cb-unc-119(+)]I; ijmSi69[Pmex-5::cls-2_reencoded::mCherry::tbb-2 3' UTR]II; unc-119(ed3)III?.

JDU447 ijmSi94 [Pmex-5::mCherry::his-11::tbb- 2 3' UTR]II; unc-119(ed3)III?; hcp-2(ijm6)V, ddIs6[gfp::tbg-1; unc-119(+)]V.

JDU464 ijmSi104[Phcp-1::gfp::hcp-1_reencoded_∆1-881; Cb-unc-119(+)]I; unc-119(ed3)III.

JDU467 ijmSi105[Phcp-1::gfp::hcp-1_reencoded_∆1-1153; Cb-unc-119(+)]I; ijmSi69[Pmex-5::cls-2_reencoded::mCherry::tbb-2 3' UTR]II; unc-119(ed3)III?.

JDU479 ijmSi106[Pmex-5::cls-2_∆CTD::gfp::hcp-1_AA1154-1386; Cb-unc-119(+)]II; it75[knl-1::mCherry]III; unc-119(ed3)III?; hcp-2(ijm6)V, ddIs6[gfp::tbg-1; unc-119(+)]V.

JDU507 ltSi259[Phcp-1::gfp::hcp-1_reencoded; Cb-unc-119(+)]I; ijmSi112[Pbub-1::bub-1_∆KD::mCherry; Cb-unc-119(+)]II; unc-119(ed3)III?.

JDU527 ijmSi7[Pmex-5::gfp::tbb-2; mCherry::his-11; Cb-unc-119(+)]I; ijmSi112[Pbub-1::bub-1_∆KD::mCherry; Cb-unc-119(+)]II; unc-119(ed3)III.

JDU572 ijmSi98[Pmex-5::mCherry::his11::tbb-2 3' UTR]II; unc-119(ed3)III?; hcp-2(ijm6)V.

JDU605 ijmSi63[Pmex-5::gfp::tba-2; mCherry::his-11; Cb-unc-119(+)]II; unc-119(ed3)III?; hcp-2(ijm6)V.

JDU631 ltSi259 [Phcp-1::gfp::hcp-1 _reencoded; Cb-unc-119(+)]I; ijmSi63[Pmex-5::gfp::tba-2; mCherry::his-11; Cb-unc-119(+)]II; unc-119(ed3)III?; hcp-2(ijm6)V, ddIs6 [gfp::tbg-1; unc-119(+)]V.

JDU632 ijmSi80[Phcp-1::gfp::hcp-1_reencoded_∆1154-1386; Cb-unc-119(+)]I; ijmSi63[Pmex-5::gfp::tba-2; mCherry::his-11; Cb-unc-119(+)]II; unc-119(ed3)III?; hcp-2(ijm6)V, ddIs6[gfp::tbg-1; unc-119(+)]V.

JDU633 ijmSi94[Phcp-1::gfp::hcp-1_reencoded_∆111-340; Cb-unc-119(+)]I; ijmSi63[Pmex-5::gfp::tba-2; mCherry::his-11; Cb-unc-119(+)]II; unc-119(ed3)III?: hcp-2(ijm6)V, ddIs6[gfp::tbg-1; unc-119(+)]V.

JDU657 ijmSi95[Pmex-5::mCherry::his11::tbb-2 3' UTR]II; unc-119(ed3)III?; hcp-2(ijm6)V, ddIs6[gfp::tbg-1; unc-119(+)]V.

JDU697 ijmSi3[Pmex-5::cls-2::reencoded::gfp::tbb-2; Cb-unc-119(+)]I; ijmSi112[Pbub-1::bub-1_∆KD::mCherry; Cb-unc-119(+)]II; unc-119(ed3)III.

JDU720 ijmSi7[Pmex-5::gfp::tbb-2; mCherry::his-11; Cb-unc-119(+)]I; ijmSi106[Pmex-5::CLS-2_reencoded_∆CTD)::gfp::HCP-1(aa1154-1386); Cb-unc-119(+)]II; unc-119(ed3)III?; hcp-2(ijm6)V.

JDU725 ijmSi3[Pmex-5::cls-2_reencoded::gfp::tbb-2 3’ UTR; Cb-unc-119(+)]I; ltSi758[Pbub-1::bub-1_D814N_reencoded::mCherry; Cb-unc-119(+)]II; unc-119(ed3)III?

JDU753 ijmSi144[Phcp-1::gfp::hcp-1_reencoded_∆aa1311-1386; Cb-unc-119(+)]I; ijmSi31[Pmex-5::mCherry::his11::tbb-2 3' UTR]II; unc-119(ed3)III.

JDU755 bub-3(ok3437)II ; unc-119(ed3)III?; hcp-1(syb441[GFP::HCP-1])V.

JDU760 ijmSi144[Phcp-1::gfp::hcp-1_reencoded_∆aa1311-1386; Cb-unc-119(+)]I; ijmSi31[Pmex-5::mCherry::his11::tbb-2 3' UTR]II; unc-119(ed3)III?; hcp-2(ijm6)V.

JDU763 ijmSi144[Phcp-1::gfp::hcp-1_reencoded_∆aa1311-1386; Cb-unc-119(+)]I; ijmSi63[Pmex-5::GFP::tba-2; mCherry::his-11; cb-unc-119(+)]II; unc-119(ed3)III?; hcp-2(ijm6)V.

PHX441 ijmSi31[Pmex-5::mCherry::his11::tbb-2 3’ UTR]II; unc-119(ed3)III?; hcp-1(syb441[gfp::HCP-1])V.

VC2773 bub-3(ok3437)II.
